# Surfactin–Bacillaene Copathway Engineering Strategy Boosts Fengycin Production and Antifungal Activity in *Bacillus velezensis* HN-Q-8

**DOI:** 10.3390/microorganisms14010246

**Published:** 2026-01-21

**Authors:** Yuzhu Gao, Liuhui Zhao, Dai Zhang, Dongmei Zhao, Qian Li, Haibin Jiang, Yang Pan, Jiehua Zhu, Zhihui Yang

**Affiliations:** 1 College of Plant Protection, Hebei Agricultural University, Baoding 071001, China; 2 Technological Innovation Center for Biological Control of Crop Diseases and Insect Pests of Hebei Province, Hebei Agricultural University, Baoding 071001, China

**Keywords:** metabolic engineering, secondary metabolites, lipopeptides, *Bacillus velezensis*, volatile organic compounds (VOCs)

## Abstract

Previous studies have demonstrated that *Bacillus velezensis* HN-Q-8 shows significant inhibitory effects against various plant pathogenic fungi causing potato diseases, primarily attributed to the production of fengycin. However, the low yield of fengycin in wild-type strains limits its practical application, and the influence of its biosynthesis pathway on volatile organic compound production remains unclear. In this study, to enhance fengycin production in *Bacillus velezensis* HN-Q-8, we applied metabolic engineering by targeting competitive pathways. Specifically, a double mutant (*ΔsrfAAΔbaeBE*) was constructed by knocking out the surfactin synthase gene *srfAA* and the bacillaene synthesis gene *baeBE*. The fengycin yield of the *ΔsrfAAΔbaeBE* mutant in the basal (sodium glutamate) fermentation medium reached 98.83 mg/L, representing a 2.39-fold increase over the wild-type strain. Subsequent medium optimization by supplementing peptone further boosted production to 155.61 mg/L, which was 3.77-fold higher than the wild-type level. The lipopeptide extract from the double mutant strain *ΔsrfAAΔbaeBE* demonstrated potentiated antifungal activity against four major potato fungal pathogens: *Alternaria solani* (early blight), *Rhizoctonia solani* (black scurf), *Fusarium oxysporum* (wilt), and *Botrytis cinerea* (gray mold). The active volatile compounds released by *ΔsrfAAΔbaeBE*, such as benzaldehyde and 2,5-dimethylpyrazine were significantly increased. The knockout of *srfAA* and *baeBE* also distinctly altered the physiology of the strain: the double mutant exhibited enhanced biofilm formation, an accelerated early growth rate followed by early decline, and a severely reduced sporulation capacity. These results confirmed the feasibility of molecularly modifying *Bacillus velezensis* HN-Q-8 to improve fengycin production and antifungal activity for further agricultural application.

## 1. Introduction

Fengycin, an environmentally friendly lipopeptide antibiotic, is a pivotal member of the lipopeptide family owing to its potent antifungal activity [1,2,3]. Produced by *Bacillus strains*, fengycin has been demonstrated to be significant efficacy against many phytopathogenic filamentous fungi, including *Monilinia laxa*, *Monilinia fructicola*, *Rhizoctonia solani*, *Fusarium moniliforme*, *Fusarium graminearum*, *Alternaria alternata*, *Alternaria solani* and *Botrytis cinerea* [4,5,6,7,8,9,10,11,12]. For instance, in potato cultivation, fengycin has been demonstrated to be a key agent in the biocontrol of early blight caused by *Alternaria solani* [12], which aligns with the focus of the present study. Its antifungal mode of action involves causing ultrastructural destruction of fungal hyphae, specifically leading to unconsolidated cytoplasm and discontinuous and/or detached cell walls from the cell membrane [3,12,13,14]. Furthermore, the critical role of fengycin in biocontrol is evidenced by the significantly reduced antifungal efficacy against Verticillium dahlia observed in the fengycin-deficient mutant *ΔFenC* of *B. subtilis* NCD-2 [15]. Despite its promising agricultural applicability, the industrial-scale production of fengycin faces significant bottlenecks. The low productivity of *Bacillus strains* has constrained the widespread application and industrialization of fengycin. Consequently, augmenting fengycin biosynthesis through molecular engineering is a critical research goal.

Molecular strategies to enhance fengycin yield in producer strains primarily target three key pathways: diverting biosynthetic precursors from competing metabolic routes, augmenting the supply of essential precursors, and overexpressing the fengycin synthesis pathway. Eliminating competing pathways redirects metabolic flux towards fengycin production and alleviates metabolic burden. For instance, knockout of the *srfAB-AC* and *pksBCDE* genes in *B. subtilis* 168 increased fengycin yield by 54% [16]. Enhancing the availability of fatty acid and amino acid precursors, such as increasing acyl-CoA pools, accelerating fatty acid biosynthesis, improving fatty acid utilization efficiency, or boosting the supply of specific amino acids, significantly elevates fengycin biosynthesis [17,18]. Specific interventions include upregulating the proline transporter gene opuE combined with 8.00 g/L exogenous proline, which raised fengycin production in *B. subtilis* 168 from 753.47 mg/L to 871.86 mg/L, and overexpressing key genes in the fatty acid pathway, which increased yield from 174.63 mg/L to 258.52 mg/L [16,19]. Transcriptional regulation is also critical. For example, replacing the native pfen promoter in the high-yielding strain *B. subtilis* BGG21 (480 mg/L) with the Ppps promoter from the low-yielding strain BBG111 (22.43 mg/L) resulted in an approximately 10-fold increase in fengycin production [9]. Optimization of fermentation conditions critically impacts fengycin synthesis by enhancing the cellular growth environment. Key strategies include modifying nitrogen sources and adjusting oxygen transfer rates. For instance, utilizing glutamate or a combination of glutamate and ammonium sulfate as nitrogen sources, alongside controlling oxygen availability through adjustments to the filling volume (e.g., 10% or 33%), has been shown to significantly enhance fengycin yield [9]. Furthermore, specific medium formulations substantially boost production; a medium containing mannitol, soybean meal, NaNO_3_, and MnSO_4_·4H_2_O increased fengycin production by *B. subtilis* F29-3 approximately 1.9-fold [20]. Targeted amino acid supplementation represents another effective approach, with lysine and alanine increasing fengycin production by 27% and 47%, respectively [21]. In this study, using metabolic engineering strategy, the surfactin synthase gene *srfAA* and bacillaene synthesis gene *baeBE* were targeted and knocked out to eliminate competitive consumption of precursor substances, as well as optimizing the composition of the culture medium to increase fengycin production.

Beyond direct fungal growth inhibition, fengycin can affect *Bacillus* biofilm formation. Furthermore, the inherent complexity of secondary metabolite networks necessitates comprehensive evaluation of engineered strains, as disruption of key genes can induce pleiotropic effects. For instance, the master regulator *Spo0A* exhibits phosphorylation-dependent functional duality, which governed sporulation and biofilm formation, while the dephosphorylated state promoted lipopeptide biosynthesis [22]. Deleting *kinA* and *rapA* significantly enhanced fengycin yield while suppressed sporulation and biofilm formation [23]. Notably, surfactin further modulated this regulatory network by activating the sporulation kinase KinC, which triggers *Spo0A* phosphorylation and downstream development of sporulation and biofilms [24]. However, there are limited reports on the effects of fengycin metabolic pathway on the synthesis of other secondary metabolites, especially volatile organic compounds (VOCs). Therefore, we conducted a comprehensive evaluation of the impact of fengycin synthesis pathways on the characteristics of the *Bacillus* strain, especially VOCs production.

In the present study, *B. velezensis* HN-Q-8, which has shown excellent biocontrol efficacy in field trials, and fengycin was identified as the main antifungal active compound secreted by *B. velezensis* HN-Q-8, which exhibited the inhibition rate up to 70–90% against plant pathogenic fungi such as *F. oxysporum*, *A. solani*, *R. solani*, and *B. cinerea* in our previous study [25]. Therefore, *B. velezensis* HN-Q-8 was targeted to engineer by disrupting the surfactin synthetase gene *srfAA* and bacillaene synthesis genes baeBE. We investigated the role of *srfAA* and *baeBE* in fengycin production and antifungal activity. In addition, their effects on VOC synthesis and the growth of *B. velezensis* HN-Q-8 were evaluated. The double knockout affected all these aspects, suggesting that targeting these competitive pathways is a viable approach to improve fengycin yield and tailor the strain’s biocontrol properties for agriculture.

## 2. Materials and Methods

### 2.1. Strains and Plasmids

All strains and plasmids used in this study are listed in Table 1. Escherichia coli DH5α was used for plasmid construction and transformation. *B. velezensis* HN-Q-8 and *E. coli* DH5α as well as their derivatives were cultured in Luria−Bertani (LB) broth (10 g/L tryptone, 5 g/L yeast extract, and 10 g/L sodium chloride, 5 mL in test tubes for routine culture; 100 mL in 250 mL flasks for seed culture) at 37 °C. When selecting for plasmids in *E. coli*, the final concentration of ampicillin was 50 μg/mL.

### 2.2. Plasmid and Mutant Strain Construction

The base plasmid PYC127 was linearized using AatII and EcoRV restriction enzymes (New England Biolabs, Beijing, China). Then, *srfAA* and *baeBE* homologous arms and the erythromycin and chloramphenicol resistance genes were amplified by PCR. The PCR was carried out with Super Kfx DNA Polymerase. The thermal cycling protocol comprised an initial denaturation step at 98 °C for 3 min; 35 cycles of denaturation at 98 °C for 10 s, annealing at 65 °C for 50 s, and extension at 72 °C for 60 s; and a final extension at 72 °C for 1 min. The seamless cloning kit (Beijing Quanshijin Biotech Co., Ltd., Beijing, China) was used to assemble the *srfAA* homologous arms and chloramphenicol resistance gene into linearized PYC127, creating the knockout plasmid PYC127-*ΔsrfAA*. Similarly, the *baeBE* homologous arms and erythromycin resistance gene were connected to linearized PYC127 via the seamless cloning kit to obtain PYC127-*ΔbaeBE* (Table 2).

The transformation of *B. velezensis* was performed chemically [26]. A single colony of HN-Q-8 from an LB agar plate was cultured overnight in LB broth at 37 °C and 200 rpm. The steps were as follows:

The overnight culture was adjusted to OD_600_ ≈ 0.3 with GCHE medium (10 mL in 50 mL tubes; 10 g/L glucose, 2 g/L potassium L-glutamate, 100 mM potassium phosphate buffer (pH = 7.0), 0.88 g/L trisodium citrate, 0.36 g/L MgSO_4_, 0.022 g/L ammonium iron citrate, 0.05 g/L L-tryptophan, 2 g/L hydrolyzed casein). Then, it was cultured to OD_600_ ≈ 1.4 at 37 °C and 200 rpm. The culture was diluted with an equal volume of GC medium (10 g/L glucose, 100 mM potassium phosphate buffer (pH7.0), 0.88 g/L trisodium citrate, 0.36 g/L MgSO_4_, 0.022 g/L ammonium iron citrate, 0.05 g/L L-tryptophan) and incubated for 1 h at 37 °C and 200 rpm. The culture was divided into 3 mL tubes, centrifuged at 6000 rpm and 4 °C for 5 min, and the pellet was resuspended in 200 μL supernatant. Then, 1 μg plasmid DNA and 2 mL transformation buffer ((NH_4_)_2_SO_4_ 1.98 g/L, K_2_HPO_4_ 13.92 g/L, KH_2_PO_4_ 6.12 g/L, MgCl_2_ 2.85 g/L, EGTA 0.38 g/L, glucose 4.5 g/L, trisodium citrate 10.29 g/L) were added. It was cultured for 30 min at 37 °C and 75 rpm. After adding 1 mL LB broth with 0.3 μg/mL erythromycin, it was cultured for 2 h at 37 °C and 200 rpm. Finally, 200 μL of the culture was spread onto solid LB agar plates with 3 μg/mL erythromycin or 5 μg/mL chloramphenicol and incubated at 37 °C. The next day, single colonies were picked and cultured overnight in liquid medium with the corresponding antibiotic. The full genome of the resulting mutants was sequenced to confirm positive clones. For the double mutant strain, the *ΔsrfAA* strain was used as the host to introduce PYC127-*ΔbaeBE*, yielding *ΔsrfAAΔbaeBE* (Table 3).

### 2.3. Extraction of Lipopeptide Compounds and Detection of Fengycin

The extraction of fengycin was performed with minor modifications of the previous method [23]. A single colony from an LB agar plate was inoculated into seed medium (100 mL seed medium in a 250 mL flask; 5 g/L beef extract, 10 g/L peptone, 5 g/L yeast extract, 5 g/L NaCl, 10 g/L glucose, pH = 7.0) and cultured for 24 h at 37 °C and 180 rpm. Then, 2% of the seed culture was transferred to 100 mL fermentation medium in a 250 mL flask fermentation medium (5 g/L sodium L-glutamate, 0.5 g/L KCl, 1 g/L KH_2_PO_4_, 0.5 g/L MgSO_4_, 5 mg/L MnSO_4_, 0.16 mg/L CuSO_4_, 0.15 mg/L FeSO_4_·7H_2_O, 20 g/L glucose, pH = 7.0) and cultured for 48 h at 37 °C and 200 rpm. The fermentation broth was transferred to a 50 mL centrifuge tube, centrifuged at 5000 rpm and 4 °C for 10 min to remove cells, and the supernatant was adjusted to pH 2.0 with 6 M HCl and left overnight at 4 °C. After re-centrifugation, the supernatant was extracted with methanol. The solution was then centrifuged at 8000 rpm and 4 °C for 10 min, and the supernatant was the crude lipopeptide extract, which was diluted to 25 mL and filtered through a 0.22 μm membrane for HPLC analysis. HPLC conditions: An Agilent ZORBAX Eclipse Plus C18 column (5 μm particle size, 250 mm × 4.6 mm i.d.; Agilent Technologies, Santa Clara, CA, USA) was used. The column temperature was maintained at 30 °C, the injection volume was 10 μL, and the mobile phase consisted of 60% acetonitrile in water (0.1% TFE) at a flow rate of 1 mL/min. Detection was performed at 210 nm [27,28]. A commercial fengycin standard (≥90.0% purity, KKL Med, Singapore) was used for calibration. The reported “fengycin yield” represents the semi-quantified total of major fengycin homologues, identified by co-elution with the standard. Representative HPLC chromatograms of the lipopeptide extracts are provided in Supplementary Figure S1.

### 2.4. Antifungal Activity Assay

Antifungal activity was evaluated using the agar diffusion method [29]. Briefly, a 5 mm mycelial plug from the actively growing edge of a 7-day-old fungal culture was placed at the center of a Potato Dextrose Agar (PDA) plate. Sterile Oxford cups (outer diameter: 8 mm) were aseptically placed on the agar surface at a distance of 25 mm from the edge of the central fungal plug. Each cup was filled with 200 μL of the filter-sterilized lipopeptide extract; an equal volume of methanol served as the negative control. The plates were incubated at 25 °C for 5 days. The width of the inhibition zone (the clear area between the edge of the Oxford cup and the edge of fungal growth) was measured in millimeters (mm) along two perpendicular diameters using a digital caliper, and the average value was calculated. The tested extracts were applied at equal volumes (200 µL) derived from equivalent fermentation culture volumes, allowing comparison of the total secreted antifungal activity per unit culture.

Lipopeptide solution was sprayed onto leaves and tubers, with distilled water and azoxystrobin as controls. After 24 h at 25 °C in the dark, spore suspensions or plugs were inoculated. Leaves were incubated at 25 °C with alternating light/dark cycles, and tubers in the dark for 5 days. Disease spot areas were then assessed.

### 2.5. Analysis of Volatile Compounds

The GC column was conditioned until the baseline was stable. The DVB/CAR/PDMS extraction fiber was also aged. Then, 5.0 mL of bacterial culture was placed in a 20 mL headspace vial, heated at 50 °C for 30 min, and extracted with the fiber for 40 min. After extraction, the fiber was immediately inserted into the GC injector for thermal desorption. Volatile compounds were separated using an HP-INNOWax capillary column (30 m length × 0.25 mm i.d. × 0.25 μm film thickness; Agilent Technologies, USA). High-purity helium was used as the carrier gas at a constant flow rate of 1.0 mL/min. The oven temperature program was set as follows: initial temperature 50 °C, increased to 240 °C at a rate of 4 °C/min, and held for 10 min. Injector temperature: 250 °C, splitless mode. EI source, 70 eV. Transfer line 250 °C, ion source 230 °C, quad pole 150 °C. Solvent delay 3 min, scan range *m*/*z* 40–550. Mass spectra were matched against the NIST 14 library, accepting hits with a Similarity Index (SI) ≥ 800 as tentative identifications (unconfirmed by standards). For comparison, compounds with a relative peak area > 1% and retention index (RI) > 800 were selected. The complete set of chromatograms (Supplementary Figure S2) and a detailed list of identified volatile compounds (Supplementary Figure S3) are provided in the Supplementary Materials.

### 2.6. Measurement of the Effects of Gene Knockout on Growth and Physiological Traits of HN-Q-8

Growth curve; The strain was activated on LB agar, a single colony was inoculated into LB broth and cultured for 24 h. Then, it was transferred to a 250 mL flask with 100 mL LB broth at a 2% inoculation rate and cultured at 37 °C and 220 rpm. Samples were taken every 6 h to measure OD_600_ and plot the growth curve.

Biofilm assay: In a 48-well plate, 1 mL LB Glycerol Manganese (LBGM) medium was added per well with 2% inoculum. After static incubation at 37 °C for 24 or 48 h, the medium was removed, and the biofilm was stained with 0.1% crystal violet. After washing and drying, 33% acetic acid was added to decolorize, and OD_570_ was measured [30].

Spore formation rate: The culture was grown in LB medium, diluted, and part was heated at 80 °C for 20 min. The spore formation rate was calculated as (heated CFU/unheated CFU) × 100%.

Adsorption capacity assay: Bacteria (OD_600_ = 1.0) were incubated in LB broth. After centrifugation and washing, the cells were resuspended and diluted. Congo red was added, and after incubation and centrifugation, the CR adsorption was calculated as (OD_490_ of blank − OD_490_ of sample) × 44.676/OD_600_ [31].

### 2.7. Data Analysis

Data are presented as the mean ± standard deviation (SD) of three independent biological replicates. Statistical significance among multiple groups was determined by one-way ANOVA followed by Tukey’s post hoc test. Differences were considered significant at *p* < 0.05, and are indicated by different lowercase letters in the figures. The sample size (*n*) for each experiment is provided in the corresponding figure legend.

## 3. Results

### 3.1. Knockout of Competitive Surfactin–Bacillaene Copathway Associated with Fengycin Synthesis

**Figure 1:**
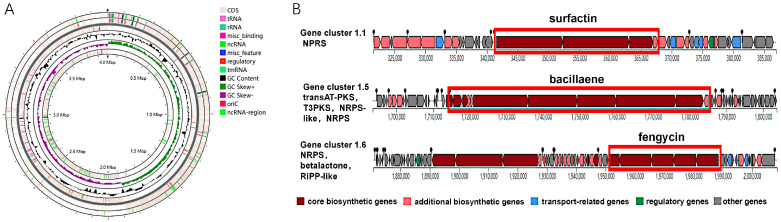
Genome analysis of *B. velezensis* HN-Q-8. (**A**) Circular genome map of HN-Q-8. (**B**) Gene clusters for surfactin, bacillaene, and fengycin synthesis.

According to the antiSMASH database annotation [32] (https://antismash.secondarymetabolites.org/ (accessed on 16 November 2024)), the genome of *B. velezensis* HN-Q-8 harbors biosynthetic gene clusters for fengycin, surfactin, and bacillaene (Figure 1A,B). Given that the synthesis of both fengycin and surfactin requires common precursors (e.g., glutamic acid, valine, and fatty acids), and considering that bacillaene biosynthesis is known to consume substantial amounts of energy and metabolic substrates, we disrupted partial sequences of the *srfAA*-AD gene cluster (responsible for surfactin production) and the *baeA-E* gene cluster (involved in bacillaene synthesis) to alleviate this metabolic competition and obtain a series of mutant strains.

**Figure 2:**
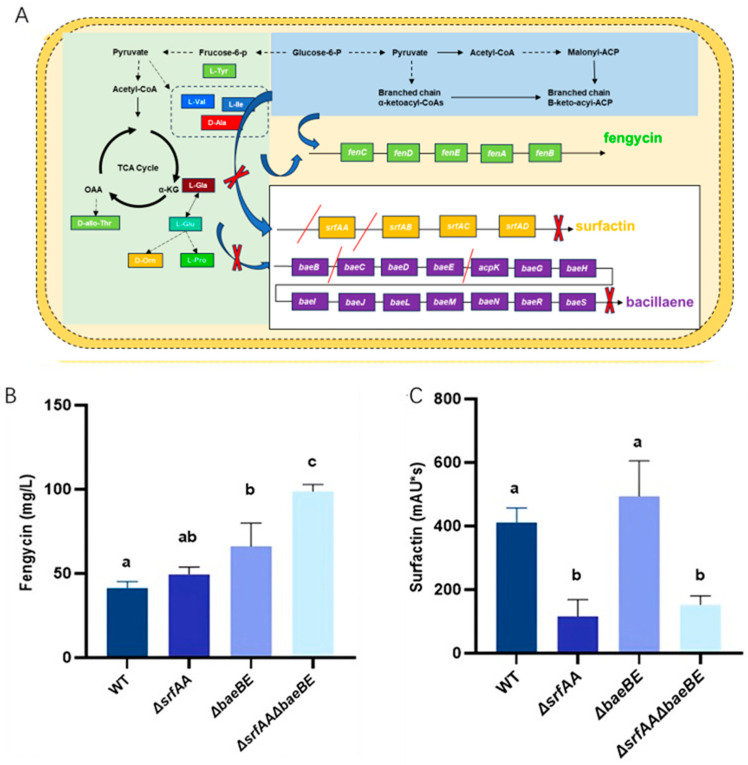
The strategy for enhancing fengycin production in *B. velezensis* HN-Q-8 and the effect of knocking out the competitive pathway on fengycin production. (**A**) Competitive relationship between fengycin, surfactin, and bacillaene. In panel (**A**), solid arrows represent direct enzymatic reactions or primary metabolic connections, dashed arrows represent simplified or multi-step pathways. The red “×” symbol indicates the blockage or inhibition of the corresponding metabolic step. Double red slashes (//) and the gene names between them indicate that the gene segment has been knocked out. (**B**) Fengycin productions of strains wild type, *ΔsrfAA*, *ΔbaeBE*, *ΔsrfAAΔbaeBE*. (**C**) Surfactin productions of strains wild type, *ΔsrfAA*, *ΔbaeBE*, *ΔsrfAAΔbaeBE*. Data are presented as means of three replicates ± SDs, and error bars represent the SDs for three replicates. Means with different letters have significant differences (*p* < 0.05).

Specifically, we inactivated the key gene *srfAA* via homologous recombination using a knockout plasmid containing flanking upstream and downstream homology arms. Subsequently, leveraging the *ΔsrfAA* background strain, we constructed a second knockout plasmid targeting the *baeB* and *baeE* genes. This deletion effectively blocked the bacillaene synthesis pathway, resulting in the generation of the double mutant strain *ΔsrfAAΔbaeBE* (Figure 2A).

As shown in Figure 2B, the knockout of surfactin synthesis alone did not significantly affect fengycin production. Notably, fengycin production was significantly enhanced in strains *ΔbaeBE* and *ΔsrfAAΔbaeBE*, reaching 66.12 mg/L and 98.83 mg/L, respectively. Furthermore, in the knockout strains *ΔsrfAA* and *ΔsrfAAΔbaeBE*, surfactin production remained low at 28% and 37% of WT, respectively (Figure 2C). Based on these results, strain *ΔsrfAAΔbaeBE* was selected for subsequent studies.

### 3.2. Effects of Different Medium Components on Fengycin Production in B. velezensis HN-Q-8

**Figure 3:**
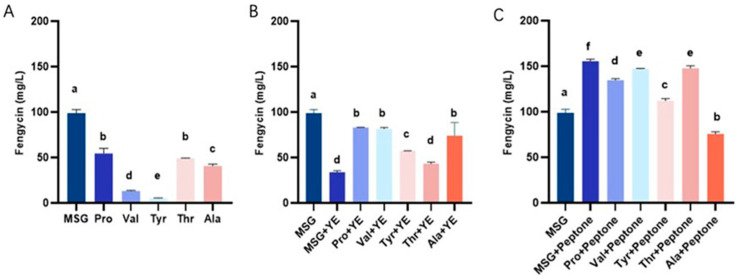
Optimization of fengycin production in *Bacillus velezensis* HN-Q-8 *ΔsrfAAΔbaeBE* through medium composition. (**A**) Effects of replacing the basal sodium glutamate with single exogenous amino acids (Pro, Val, Tyr, Thr, Ala) on fengycin yield. (**B**) Fengycin yield in media containing both an exogenous amino acid and yeast extract (YE). (**C**) Fengycin yield in media containing both an exogenous amino acid and peptone. MSG: monosodium glutamate (basal control). Data are mean ± SD (*n* = 3). Different lowercase letters indicate statistically significant differences among treatments (*p* < 0.05, one-way ANOVA with Tukey’s HSD test).

To further optimize fengycin production in the double mutant strain *ΔsrfAAΔbaeBE*, we investigated the effects of supplementing the fermentation medium with five amino acids, including proline, valine, tyrosine, threonine, and alanine. As shown in Figure 3A, when cultivated in proline medium, *ΔsrfAAΔbaeBE* produced 54.47 mg/L fengycin. Strikingly, in sodium glutamate medium, fengycin production in *ΔsrfAAΔbaeBE* reached 98.83 mg/L. We further investigated the impact of supplementing nitrogen sources with yeast extract powder or peptone on fengycin production. The combination of proline with yeast extract yielded the highest among these groups, but remained significantly lower than the production achieved with sodium glutamate alone. However, supplementing with 10 g/L peptone significantly enhanced fengycin production (Figure 3B). Notably, in the optimized medium containing sodium glutamate plus peptone, the fengycin yield of *ΔsrfAAΔbaeBE* reached 155.61 mg/L. This represented a 1.57-fold increase over its production in the basal sodium glutamate medium (98.83 mg/L) and a 3.77-fold increase over the wild-type strain. (Figure 3C). These results demonstrate that optimizing the nitrogen source composition in the culture medium significantly enhances fengycin production, providing crucial insights for subsequent fermentation optimization.

### 3.3. Antifungal Activity of Fengycin Produced by B. velezensis HN-Q-8

**Figure 4:**
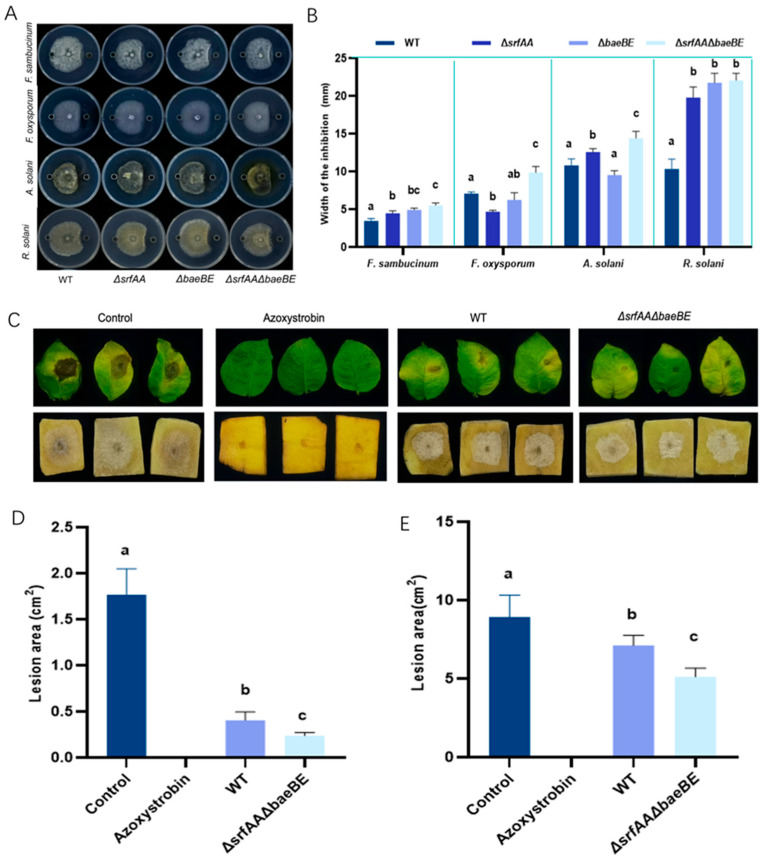
In vitro and in vivo antifungal activity of lipopeptide extracts from *Bacillus velezensis* HN-Q-8 and its mutant strains (**A**,**B**) In vitro inhibition zones against *Alternaria solani* (**A**) and Rhizoctonia solani (**B**). (**C**,**D**) In vivo protective effect against *A. solani* on detached potato leaves: representative disease symptoms (**C**) and quantified lesion areas (**D**). (**E**) In vivo protective effect against *R. solani* on potato tuber slices: representative disease symptoms (left panel) and quantified lesion areas (right panel). WT: wild-type strain HN-Q-8; *ΔsrfAA*: surfactin synthase gene mutant; *ΔbaeBE*: bacillaene synthesis gene mutant; *ΔsrfAAΔbaeBE*: double mutant. Data in bar graphs (D and E right panel) are presented as mean ± standard deviation (*n* = 3 independent experiments). Different lowercase letters above bars indicate statistically significant differences (*p* < 0.05) as determined by one-way ANOVA followed by Tukey’s HSD test.

Fengycin, an antifungal lipopeptide, exhibits specific inhibitory activity against filamentous fungi. Antifungal assays demonstrated that lipopeptides produced by mutant strains exhibited enhanced inhibition against four fungi. The double mutant *ΔsrfAA ΔbaeBE* produced the largest inhibition zones among all strains tested. Specifically, against *R. solani*, its inhibition zone (11.03 mm) was more than twice that of the wild-type strain (5.0 mm; Figure 4A,B).

In this study, *A. solani* and *R. solani* served as indicators to assess the antifungal activity of fengycin extracts from recombinant *B. velezensis* strains in vivo. As shown in Figure 4C–E, methanol exhibited no inhibitory activity against these fungi. The lipopeptides extract of *ΔsrfAA ΔbaeBE* showed significant control efficiency. Through molecular modification, we achieved enhanced fengycin production, and the resulting antifungal activity increased proportionally with fengycin concentration. These results collectively underscore the potential of fengycin as a biocontrol agent, supporting its development for agricultural and preservative applications.

### 3.4. Effects of srfAA and baeBE on the Synthesis of VOCs in B. velezensis HN-Q-8

**Figure 5:**
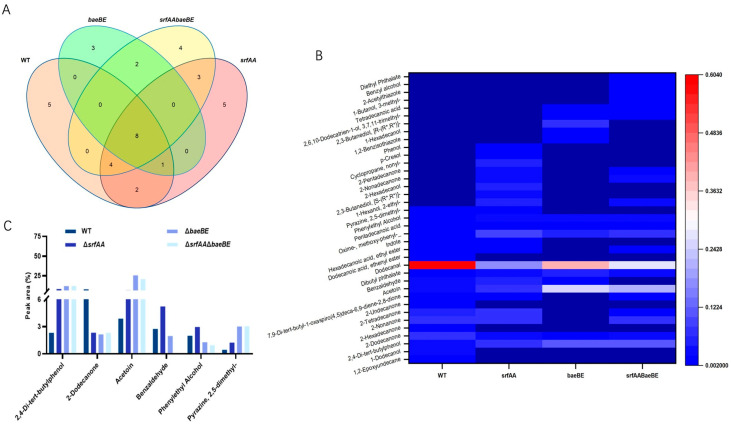
Impact of *srfAA* and *baeBE* gene knockout on the volatile metabolome of *Bacillus velezensis* HN-Q-8. (**A**) Venn diagram showing differences in the composition of volatile compounds among strains. (**B**) Heatmap depicting relative abundance (peak area) differences in volatile compounds. (**C**) Relative content changes of six volatile compounds with reported antifungal activity. Data in (**C**) are presented as mean ± SD (*n* = 3–4). WT: wild-type strain; *ΔsrfAA*: surfactin synthase gene mutant; *ΔbaeBE*: bacillaene synthesis gene mutant; *ΔsrfAAΔbaeBE*: double mutant.

Synthetic networks of secondary metabolites exhibit high complexity, where deletion of a key gene can redirect precursors or intermediates into alternative branch pathways. Consequently, we examined the impact of *srfAA* and *baeBE* knockouts on VOCs production in *B. velezensis* HN-Q-8. GC-MS analysis revealed that eight volatiles were detected in both wild-type and mutant strains. The relative levels of selected metabolites are summarized in Table 4. The wild-type and mutant strains produced distinct sets of volatile compounds. Compared to the wild-type, the *ΔsrfAA*, *ΔbaeBE*, and *ΔsrfAAΔbaeBE* mutants produced 8, 5, and 9 unique volatiles, respectively. The fact that the *srfAA* single knockout resulted in more unique volatiles than the *baeBE* knockout suggests that the *srfAA* gene has a broader influence on the volatile metabolome of *B. velezensis* HN-Q-8. (Figure 5A) These deletions also significantly altered the concentrations of volatiles (Figure 5B). Notably, benzaldehyde, 2,4-di-tert-butylphenol, acetylacetone, and 2,5-dimethylpyrazine, which have been identified as the key antifungal VOCs in previous studies, showed dramatic increases in *ΔsrfAAΔbaeBE*. Their peak areas increased by 90.88%, 412.93%, 438.40%, and 509.91%, respectively, relative to the wild-type strain (Figure 5C). These results indicated that *srfAA* and *baeBE* had a certain impact on the synthesis of these critical active volatiles.

### 3.5. Effects of srfAA and baeBE on the Physiology in B. velezensis HN-Q-8

**Figure 6:**
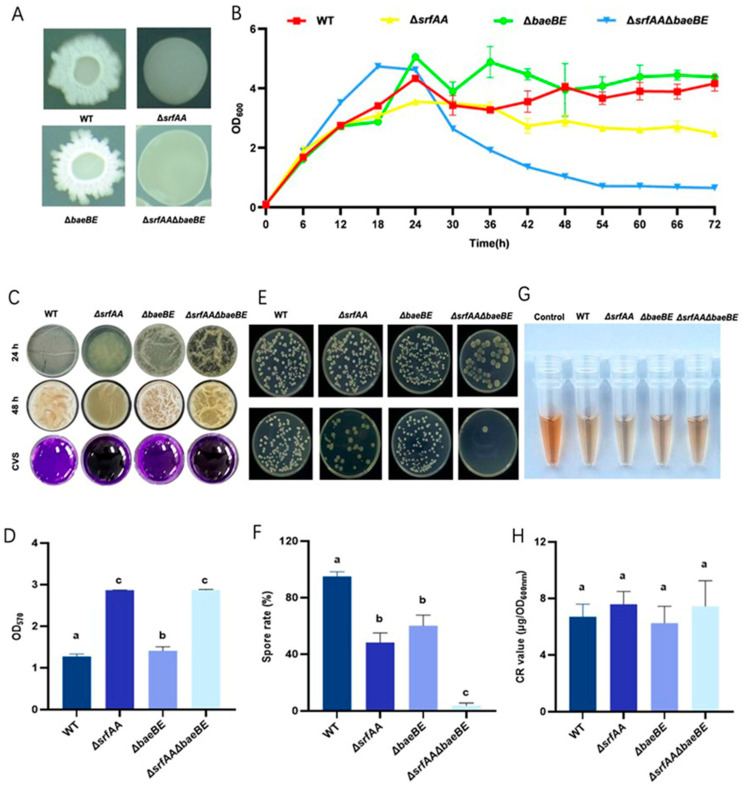
Effects of *srfAA* and *baeBE* on HN-Q-8 growth and physiology. (**A**) Representative colony morphology (1–2 cm in diameter) on LB agar plates after 48 h. (**B**) Growth curves in LB broth monitored by OD_600_. (**C**) Representative images of pellicle biofilms formed at the air-liquid interface after static incubation in LBGM medium for 24 h and 48 h. (**D**) Quantitative analysis of biofilm biomass at 48 h by crystal violet staining (OD_570_). (**E**) Visualization of heat-resistant spore formation by plating serial dilutions of cultures with or without heat treatment (80 °C, 20 min). (**F**) Sporulation efficiency calculated as the ratio of heat-resistant CFU to total CFU. (**G**) Representative tubes after Congo red (CR) adsorption assay. (**H**) Quantitative CR adsorption capacity. Data in (**B**,**D**,**F**,**H**) are presented as mean ± SD (*n* = 3). Different lowercase letters above bars in (**D**,**F**,**H**) indicate statistically significant differences among strains (*p* < 0.05, one-way ANOVA with Tukey’s HSD test). WT: wild-type strain; *ΔsrfAA:* surfactin synthase gene mutant; *ΔbaeBE*: bacillaene synthesis gene mutant; *ΔsrfAAΔbaeBE*: double mutant.

In order to evaluate the effects of *srfAA* and *baeBE* genes on the growth characteristics of bacterial strains and facilitate subsequent research and application, we further conducted studies on colony morphology, growth rate, biofilm formation, sporulation, and adsorption. As shown in Figure 6A, the colony of the *ΔbaeBE* mutant exhibited significantly more irregular edges compared to the wild-type strain. Furthermore, both *ΔsrfAA* and *ΔbaeBEΔsrfAA* displayed similar, smooth, and non-wrinkled colony morphologies, while a difference in color and opacity was observed between *ΔsrfAA* and *ΔsrfAAΔbaeBE*. To further assess the impact of *srfAA* and *baeBE* on strain growth, 72 h growth characteristics were investigated (Figure 6B). Similarly to WT, both *ΔsrfAA* and *ΔbaeBE* exhibited no significant change in growth rate, reaching their maximum OD_600_ value at 24 h. However, the *ΔbaeBEΔsrfAA* double mutant reached its peak OD_600_ value much earlier, at only 18 h. Following this peak, its OD_600_ value declined steadily. These results demonstrated that gene deletion significantly altered the growth of HN-Q-8.

The simultaneous deletion of *srfAA* and *baeBE* enhanced biofilm formation (Figure 6C,D). At 24 h and 48 h, biofilms of *ΔbaeBE* and *ΔsrfAAΔbaeBE* displayed pronounced wrinkling, while *ΔsrfAA* formed intact biofilms but showed minimal wrinkling. However, quantitative crystal violet staining of 48 h biofilms revealed an OD_570_ value of 2.87 for *ΔsrfAA* (2.26-fold higher than WT). In summary, deletion of *srfAA* reduced biofilm wrinkling but significantly increased overall biofilm biomass. The loss of surfactin and bacillaene production significantly impaired *Bacillus* sporulation capacity. As shown in Figure 6E,F, WT strain exhibited a sporulation rate of 95.03%. In contrast, the *ΔsrfAA* mutant displayed a significantly reduced sporulation rate of 48.22%, while the *ΔbaeBE* mutant showed a rate of 60.11%. Notably, the sporulation rate of the *ΔbaeBEΔsrfAA* double mutant was further diminished to only 3.74%. These results demonstrated that both *srfAA* and *baeBE* genes played critical roles in sporulation in *Bacillus* strain.

The adsorption capacity of *Bacillus strains*, referring to their ability to adsorb environmental contaminants like heavy metal ions and organic compounds, holds significant importance for environmental remediation and biosorption applications. This study assessed changes in adsorption capacity using Congo red binding assays. As shown in Figure 6G,H, no significant differences in adsorption capacity were observed among any of the mutant strains.

## 4. Discussion

Previous studies have demonstrated that *B. velezensis* HN-Q-8 showed great control efficiency on several potato diseases, with the prevention efficacy against potato powdery scab reaching 75.42% in field trials. Meanwhile, fengycin was identified as the main antifungal active compound secreted by *B. velezensis* HN-Q-8, which exhibited the inhibition rate up to 70–90% against plant pathogenic fungi such as *F. oxysporum*, *A. solani*, *R. solani*, and *B. cinerea* [25]. Therefore, we selected HN-Q-8 strain to improve fengycin production. However, due to low yields produced by *Bacillus*, industrialization of fengycin is severely constrained. In this study, the *srfAA* and *baeBE* were knocked out to eliminate competitive consumption of precursor substances using metabolic engineering strategy, and further to optimize the composition of the culture medium. Moreover, the study evaluated the changes in growth, biofilm formation, sporulation, and adsorption of mutant strains. The effects on the synthesis of other secondary metabolites, especially VOCs production, were also evaluated. The results demonstrated that the engineered strain exhibited not only significantly enhanced fengycin production and antifungal activity but also distinct alterations in colony morphology, growth rate, biofilm formation, and sporulation. The production of active VOCs was also increased significantly. These findings validate the feasibility of the HN-Q-8 modification strategy and provide a theoretical basis for further improvement and application.

The synthesis of both bacillaene and surfactin competitively consumes essential precursors such as amino acids and fatty acids, thereby limiting fengycin production efficiency [33]. To address this, we constructed a surfactin and bacillaene double mutant (*ΔsrfAAΔbaeBE*), which achieved a fengycin yield of 98.83 mg/L, representing a 2.39-fold improvement over the wild-type strain. Similarly, constructing this double mutant in *B. subtilis* 168 increased fengycin yield from 1.81 mg/L to 2.79 mg/L, a 1.54-fold enhancement [16]. The differences in yield improvement are likely due to variations between strains and their baseline production capacities, highlighting the potentially greater developmental value of strain HN-Q-8. The results indicated that nutrient supply significantly boosted fengycin production in *B. velezensis* HN-Q-8. While amino acids serve as primary substrates for fengycin synthesis, their impact on yield varies considerably across strains [34,35,36]. For instance, adding threonine and proline to *B. subtilis* 168 cultures increased fengycin production by 76.12% and 49.96%, respectively [19]. Conversely, supplementing *B. amyloliquefaciens* fermentation media with exogenous amino acids can decrease fengycin yield [23]. In our investigation, sodium glutamate was identified as the optimal amino acid supplement among six tested amino acids. Notably, the addition of peptone to the culture of the double mutant *ΔsrfAAΔbaeBE* resulted in a fengycin production of 155.61 mg/L—1.57 times the level produced in the basal medium.

Fengycin is synthesized via the nonribosomal peptide synthetase (NRPS) pathway and primarily acts by disrupting fungal membrane integrity [37]. Upon binding, fengycin molecules aggregate into large complexes, disordering membrane phospholipids, causing cytoplasmic leakage, and ultimately leading to cell death [38]. Our results confirm that *ΔsrfAAΔbaeBE* exhibits significantly enhanced inhibition against four major plant pathogens. While prior research often focused solely on plate confrontation assays without evaluating biocontrol efficacy, our tests demonstrated that *ΔsrfAAΔbaeBE* increased control efficacy against potato early blight and black scurf disease by 12.49% and 109.36%, respectively, in vivo. This not only validated the strain engineering approach but also provided a basis for field applications.

To assess the application potential of the engineered *Bacillus strains*, we evaluated growth rate, biofilm formation, sporulation, and adhesion capability. Regarding growth, bacillaene synthesis demands substantial substrate and energy resources. Consequently, although the *ΔbaeBE* single mutant did not exhibit an accelerated growth rate, its OD_600_ during the stationary phase (24–48 h) exceeded that of the wild-type. Mutations in *srfAA* can prevent relief from carbon catabolite repression upon glucose depletion, leading to cell death; viability is restored by glucose replenishment [39]. Consistent with this, we observed an OD_600_ decline in the *ΔsrfAA* mutant at 42 h, preliminarily attributed to glucose exhaustion. This effect was more pronounced in the double mutant *ΔsrfAAΔbaeBE*, which accelerated growth culminated in a peak OD_600_ by 18 h, followed by a decline starting at 24 h due to premature glucose depletion. *Bacillus* species typically colonize plant roots as cell clusters embedded within self-produced extracellular matrices [40], forming biofilms essential for spatial competition and disease suppression [31,41,42,43,44]. While surfactin has been reported to induce biofilm formation [45], we observed significantly enhanced biofilm in the double mutant *ΔsrfAAΔbaeBE*, suggesting a divergent effect. In terms of sporulation, surfactin acts as a signal molecule activating the sporulation kinase *KinC*, which induces *Spo0A* phosphorylation to promote sporulation [24]. Consistent with previous findings, *ΔsrfAA* mutant exhibited a 49.26% reduction in sporulation efficiency in this study. While bacillaene’s role in sporulation has not been previously reported, *ΔbaeBE* mutant showed a 36.75% decrease. The double mutant *ΔsrfAAΔbaeBE* displayed a drastic 96.06% reduction in sporulation. As higher sporulation rates can impede secondary metabolite production during fermentation [46], the low sporulation phenotype of *ΔsrfAAΔbaeBE* likely favors fengycin synthesis. Adhesion capability, assessed within the range of 6.20–7.60 μg/OD_600_, remained unaffected by the mutations. Collectively, these evaluations indicate that the double mutant *ΔsrfAAΔbaeBE* is more suitable for development as a fermentation-based biocontrol agent, enabling the extraction and agricultural application of its antimicrobial compounds.

Secondary metabolite networks are highly complex, and disrupting key genes often diverts precursors or intermediates into branch pathways. The effect of the fengycin metabolic pathway on volatiles production is poorly understood. In this study, analysis of VOCs revealed significant accumulation of metabolites like benzaldehyde and methoxy-phenyl-oxime in the *ΔsrfAA* mutant. The deletion of *srfAA* may activate alternative biosynthetic pathways through metabolic flux redistribution. For example, deleting *degU* enhances phosphate and amino acid transport while diminishing monosaccharide, oligosaccharide, polyol, and zinc ion transport, weakening bacillomycin D and fengycin synthesis but strengthening surfactin production [22]. Although the roles of *srfAA* and *baeBE* in VOC biosynthesis remain poorly understood, this study lays the groundwork for future research on volatile metabolite biosynthesis.

## 5. Conclusions

In this study, knockout of the *srfAA* and *baeBE* genes yielded a double mutant *ΔsrfAAΔbaeBE* with significantly enhanced fengycin production, while production further amplified to 155.61 mg/L (3.77× wild-type) in a peptone-sodium glutamate medium. The double mutant displayed stronger antifungal activity against four key fungal pathogens. These genes altered volatile compound synthesis and influenced bacterial growth, biofilm formation, and sporulation, while not affecting adhesion.

## Figures and Tables

**Table 1:** Bacteria strains and plasmids used in this experiment.

Strains and Plasmids	Origin/Key Feature	Purpose in This Study
*B. velezensis* HN-Q-8	Wild-type, isolated from potato rhizosphere [25]	Wild-type strain
*B. velezensis* HN-Q-8-*ΔsrfAA*	*srfAA* knockout of HN-Q-8 (this study)	Surfactin mutant strain
*B. velezensis* HN-Q-8-*ΔbaeBE*	*baeBE* knockout of HN-Q-8 (this study).	Bacillaene mutant strain
*B. velezensis* HN-Q-8-*ΔsrfAAΔbaeBE*	*srfAA baeBE* double knockout of HN-Q-8 (this study)	Double mutant strain
PYC127	Expression vector	Knockout vector backbone
*E. coli* DH5α	Competent cells	Cloning host

**Table 2:** Primer names and sequences.

Primer Names	Sequences (5′–3′)
bae-up-F	GAAAAGTGCCACCTGACGTATGGATCATACATATGAAGTGCATC
bae-up-R	ATACTGCACTATCAACACACTAGCCATTCATCACCAGAAATC
ErR-F	GATTTCTGGTGATGAATGGCTAGTGTGTTGATAGTGCAGTAT
ErR-R	CATGAGACTGTAAGCAACTCTCCCGATACAAATTCCCCGT
bae-down-F	ACGGGGAATTTGTATCGGGAGAGTTGCTTACAGTCTCATG
bae-down-R	CGCCCAGCCTAAACGGATTCAACACGTTTGCAAAAATGAAC

**Table 3:** Summary of key culture conditions for different experimental stages.

Experimental Stage	Medium	Temperature	Agitation (rpm)	Key Purpose
Seed culture	Seed medium	37 °C	180	Inoculum preparation
Fermentation	Fermentation medium	37 °C	200	Fengycin production
Growth curve	LB broth	37 °C	220	Growth kinetics
Biofilm formation	LBGM medium	37 °C	Static (0 rpm)	Biofilm assay
VOC collection	LB broth	37 °C	200	VOC production

**Table 4:** GC-MS analysis of selected metabolites in fermentation broth of *B. velezensis* HN-Q-8 and its mutant strains.

Compound Name	Peak Area (×10^6^, Mean ± SD)			
	WT	*ΔsrfAA*	*ΔbaeBE*	*ΔsrfAAΔbaeBE*
Benzaldehyde	0.463 ± 0.104	0.599 ± 0.021	0.197 ± 0.137	0
2,4-Di-tert-butylphenol	0.407 ± 0.068	0.922 ± 0.800	0.541 ± 0.210	1.035 ± 0.581
2,5-Dimethylpyrazine	0.074 ± 0.032	0.142 ± 0.050	0	0
Acetoin	0.655 ± 0.180	0.750 ± 0.190	2.549 ± 0.147	1.812 ± 0.130
2-Nonanone	1.033 ± 0.598	0.770 ± 0.060	0.315 ± 0.217	0.615 ± 0.030
Dodecanal	10.141 ± 3.595	2.042 ± 0.024	3.547 ± 1.672	2.435 ± 0.376
Indole	0.154 ± 0.043	0.068 ± 0.010	0.071 ± 0.009	0.040 ± 0.010

## Data Availability

The original contributions presented in this study are included in the article and Supplementary Materials. Further inquiries can be directed to the corresponding authors.

## Supplementary Materials

The following supporting information can be downloaded at: https://www.mdpi.com/article/10.3390/microorganisms14010246/s1, Figure S1: HPLC chromatogram (separation and detection of fengycin in different media); Figure S2: MS spectrum (comparative profiling of volatile metabolites in wild-type and mutant strains); Figure S3: GC-MS analysis results (identification of volatile metabolites).

## Abbreviations

The following abbreviations are used in this manuscript:

VOCs	Volatile organic compounds
HPLC	High-performance liquid chromatography
GC-MS	Gas chromatography–mass spectrometry
NRPS	Nonribosomal peptide synthetase
TFA	Trifluoroacetic acid
NIST	National Institute of Standards and Technology
antiSMASH	Antibiotics and Secondary Metabolite Analysis Shell
