# Supplementary material for: Surfactin–Bacillaene Copathway Engineering Strategy Boosts Fengycin Production and Antifungal Activity in Bacillus velezensis HN-Q-8
Source: Microorganisms. 2026 Jan 21;14(1):246. doi: 10.3390/microorganisms14010246 (PMC12843716; doi:10.3390/microorganisms14010246)
Supplement: Supplementary file 1 [file microorganisms-14-00246-s001.zip › microorganisms-4092615-supplementary.pdf]

Figure S1. HPLC chromatogram (separation and detection of fengycin in different media)

fengycin (50μg/mL)

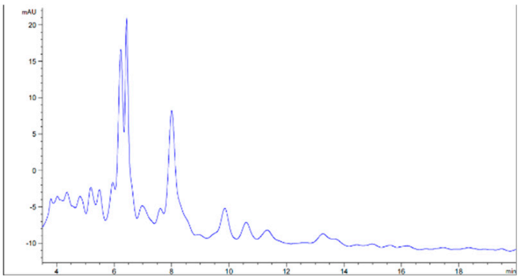

B. velezensis HN-Q-8 (Wild-type, WT) Left: LB medium; Right: Optimized medium.

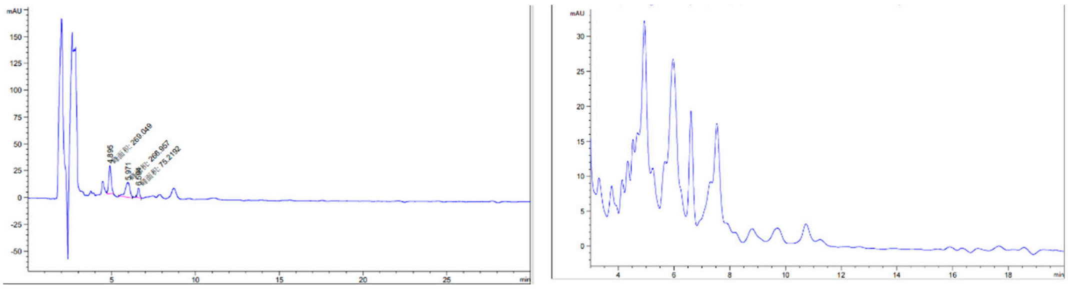

HN-Q-8-ΔsrfAA Left: LB medium; Right: Optimized medium.

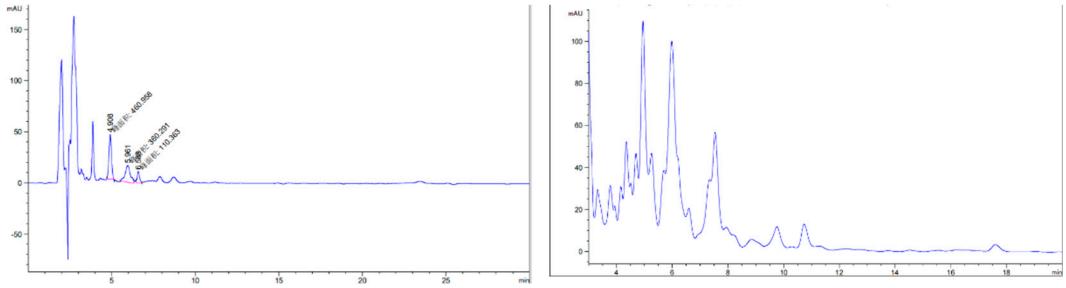

HN-Q-8-ΔbaeBE Left: LB medium; Right: Optimized medium.

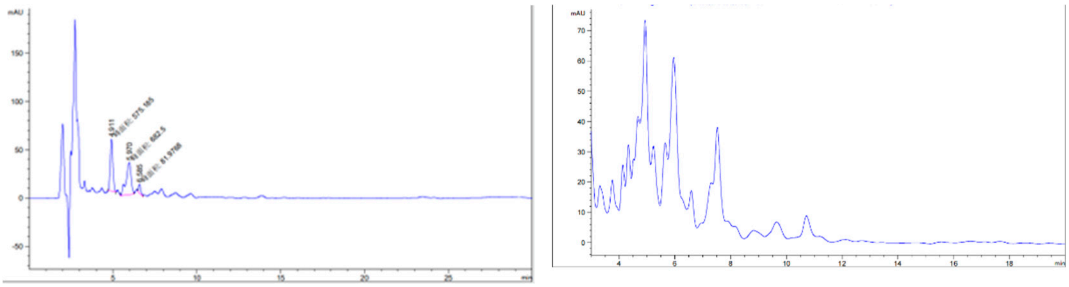

HN-Q-8-ΔsrfAAΔbaeBE Left: LB medium; Right: Optimized medium.

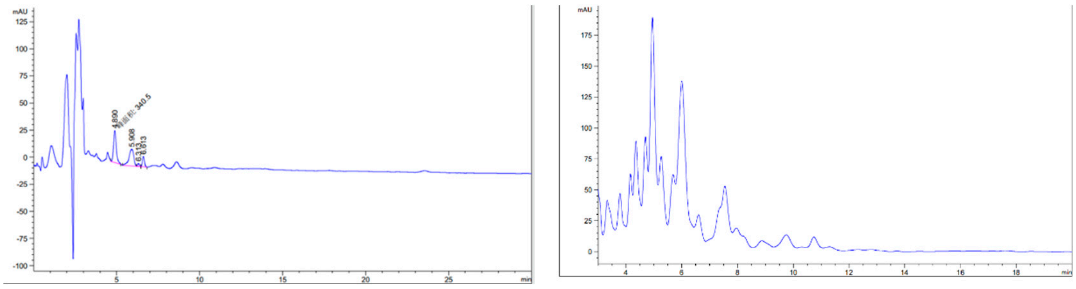

Figure S2.MS spectrum (comparative profiling of volatile metabolites in wild-type and mutant strains)

B. velezensis HN-Q-8 (Wild-type, WT)

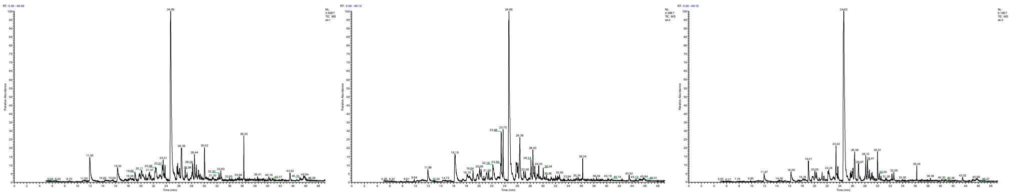

HN-Q-8-ΔsrfAA

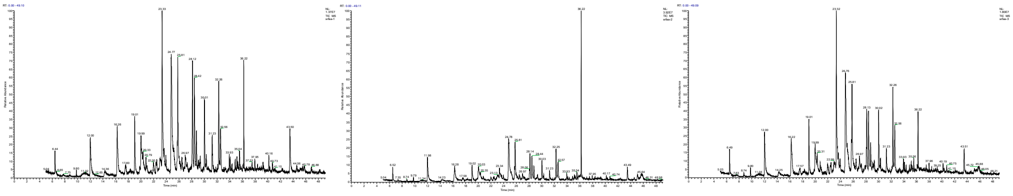

HN-Q-8-ΔbaeBE

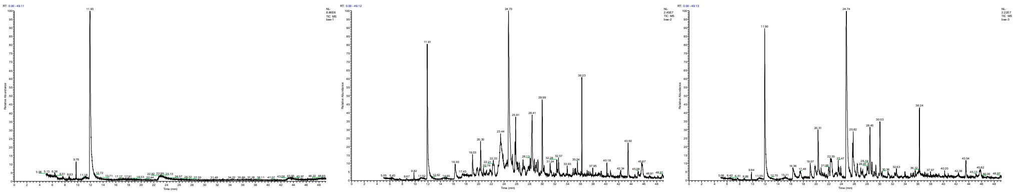

HN-Q-8-ΔsrfAAΔbaeBE

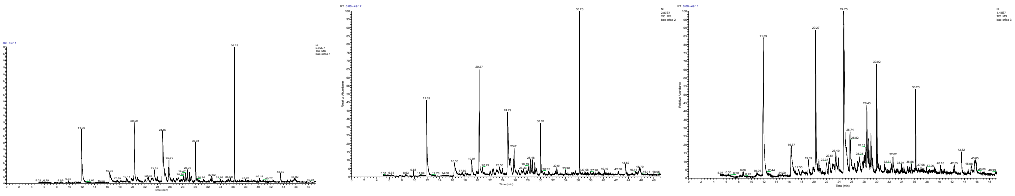

Figure S3 GC-MS analysis results (identification of volatile metabolites)

WT-1

Peak	Ret.Time	match	R.match	Prob	CAS Number	Library Compound	Area
1	8.7	806	877	50.22	123-51-3	1-Butanol, 3-methyl-	54186.64
2	11.93	854	856	79.51	513-86-0	Acetoin	869002.8
4	14.35	713	871	44.86	123-32-0	Pyrazine, 2,5-dimethyl-	15791.67
9	14.47	756	871	56.3	123-32-0	Pyrazine, 2,5-dimethyl-	97145.5
11	16.32	923	942	91.32	821-55-6	2-Nonanone	615527.6
24	19.06	936	949	67.58	104-76-7	1-Hexanol, 2-ethyl-	224469.8
25	19.24	622	773	18.82	58175-57-8	2-Propyl-1-pentanol	22721.47
27	20.11	869	931	53.26	100-52-7	Benzaldehyde	346845
28	20.36	776	926	62.51	19132-06-0	2,3-Butanediol, [S-(R*,R*)]-	192967.8
31	21.24	813	839	8.59	69064-37-5	trans-2-Dodecen-1-ol	173202.2
32	21.37	866	889	6.15	629-76-5	n-Pentadecanol	133786.4
33	21.57	779	817	11.94	13980-12-6	cis-9,10-Epoxyoctadecan-1-ol	115977.9
36	22.21	862	880	80.49	112-12-9	2-Undecanone	137587.4
37	22.34	608	877	17.2	112-44-7	Undecanal	184302.6
38	22.38	628	842	18.45	17322-97-3	1,2-Epoxyundecane	381795.7
44	23.37	753	849	73.08	98-00-0	2-Furanmethanol	346061.3
45	23.51	818	865	55.31	6175-49-1	2-Dodecanone	272079.8
46	23.64	723	864	5.79	112-54-9	Dodecanal	120215.5
47	23.8	826	832	49.92	6175-49-1	2-Dodecanone	226635.8
49	24.69	894	954	42.69	112-54-9	Dodecanal	7291919
51	25.7	876	901	9.91	112-42-5	1-Undecanol	220317.4
52	25.81	815	828	85.4	0	Oxime-, methoxy-phenyl-_	266515.7
54	26.07	852	911	13.49	112-44-7	Undecanal	292132.8
56	26.38	832	852	12.75	30689-78-2	(R)-(-)-(Z)-14-Methyl-8-hexadecen-1-ol	1130913
61	28.05	898	917	38.93	100-51-6	Benzyl alcohol	162019.3
62	28.16	812	855	54.51	2345-27-9	2-Tetradecanone	346451.6
63	28.44	765	845	38.27	2345-27-9	2-Tetradecanone	607640.3
64	28.75	896	905	########	60-12-8	Phenylethyl Alcohol	313944.3
68	29.58	763	829	38.28	2146-71-6	Dodecanoic acid, ethenyl ester	70779.73
69	30.02	918	925	11.27	112-53-8	1-Dodecanol	619746.6
72	30.36	692	801	33.69	2345-28-0	2-Pentadecanone	70025.56
76	31.3	742	784	54.59	2345-28-0	2-Pentadecanone	57780.13
78	32.14	745	857	22.74	106-44-5	p-Cresol	81699.73
79	32.33	828	837	50.91	18787-63-8	2-Hexadecanone	126775.5
81	32.6	769	822	35.43	18787-63-8	2-Hexadecanone	266358.4
87	35.35	620	745	28.31	5381-92-0	Benzeneethanol, α-(phenylmethyl)-	51148.57
88	35.58	745	796	56.38	628-97-7	Hexadecanoic acid, ethyl ester	65313.47
92	36.23	913	914	55.96	96-76-4	2,4-Di-tert-butylphenol	489127.7
98	38.41	829	890	33.38	120-72-9	Indole	107339.1
107	43.52	912	918	23.6	84-74-2	Dibutyl phthalate	217609.7
108	44.59	693	806	76.33	82304-66-3	7,9-Di-tert-butyl-1-oxaspiro(4,5)deca-6,9-die	38157.52
109	45.1	754	805	58.36	1002-84-2	Pentadecanoic acid	77093.77

WT-2

Peak	Ret.Time	match	R.match	Prob	CAS Number	Library Compound	Area
1	8.61	813	879	45.78	123-51-3	1-Butanol, 3-methyl-	62648.97
3	10.23	760	838	74.97	928-68-7	2-Heptanone, 6-methyl-	27751.82
5	10.29	639	802	57.45	928-68-7	2-Heptanone, 6-methyl-	54866.97
11	11.98	855	860	72.35	513-86-0	Acetoin	537048
12	14.31	768	857	52.85	123-32-0	Pyrazine, 2,5-dimethyl-	59921.67
14	14.72	598	807	37.95	821-55-6	2-Nonanone	66730.5
15	16.19	945	951	92.04	821-55-6	2-Nonanone	663219.5
16	16.24	915	940	90.59	821-55-6	2-Nonanone	1128698
21	18.1	712	809	53.39	693-54-9	2-Decanone	359923.3
22	18.44	740	787	47	693-54-9	2-Decanone	289149.4
23	19.04	933	946	69.77	104-76-7	1-Hexanol, 2-ethyl-	316354.7
24	19.7	798	853	35.16	112-31-2	Decanal	166625.5
26	20.06	790	917	33.52	100-52-7	Benzaldehyde	494279.4
27	20.3	844	911	64.59	24347-58-8	2,3-Butanediol, [R-(R*,R*)]-	408042.6
30	21.19	827	863	9	112-44-7	Undecanal	407064.3
34	22.16	895	898	83.77	112-12-9	2-Undecanone	349218.6
35	22.27	727	865	39.25	17322-97-3	1,2-Epoxyundecane	452750.7
37	23	729	838	15.43	0	2,4,6-Cycloheptatrien-1-one, 4-methyl-	97505.95
41	23.36	654	809	42.31	4412-91-3	3-Furanmethanol	314221.2
42	23.46	863	865	58.68	6175-49-1	2-Dodecanone	1014542
44	23.75	814	814	41.34	6175-49-1	2-Dodecanone	990377.1
45	24.66	895	961	40.71	112-54-9	Dodecanal	8809212
48	25.81	827	846	87.31	0	Oxime-, methoxy-phenyl-_	338187.7
50	26.04	840	893	11.04	75853-51-9	13-Methyltetradecanal	229862
51	26.1	838	875	17.4	10203-28-8	2-Dodecanol	357621.8
52	26.38	808	815	1900/1/7	2765-11-9	Pentadecanal-	1551813
53	27	719	861	46.63	593-08-8	2-Tridecanone	368373
58	28.04	923	940	46.43	100-51-6	Benzyl alcohol	87036.39
59	28.14	847	870	58.9	2345-27-9	2-Tetradecanone	482528.3
60	28.43	791	845	32.13	629-66-3	2-Nonadecanone	833130.5
62	28.73	929	940	########	60-12-8	Phenylethyl Alcohol	329606.3
66	29.55	768	816	69.63	2146-71-6	Dodecanoic acid, ethenyl ester	73908.26
68	29.83	684	881	32.06	272-16-2	1,2-Benzisothiazole	40941.41
69	30.04	906	916	8.23	74663-85-7	Cyclopropane, nonyl-	245171.9
71	30.31	716	821	9.85	4706-81-4	2-Tetradecanol	80104.19
73	31.28	795	818	58.43	2345-28-0	2-Pentadecanone	78984.46
80	32.13	787	866	23.57	108-39-4	Phenol, 3-methyl-	54829.88
81	32.31	808	823	51.24	18787-63-8	2-Hexadecanone	89961.27
83	32.6	778	827	36.29	18787-63-8	2-Hexadecanone	185330.3
90	35.57	719	762	45.56	628-97-7	Hexadecanoic acid, ethyl ester	74664.75
94	36.24	900	904	54.63	96-76-4	2,4-Di-tert-butylphenol	369141.1
95	37.06	720	825	12.22	3790-71-4	2,6,10-Dodecatrien-1-ol, 3,7,11-trimethyl-, (Z	34514.71
97	37.67	648	828	26.3	496-16-2	Benzofuran, 2,3-dihydro-	31752.16
99	38.39	822	876	34.78	120-72-9	Indole	162155.9
109	43.52	930	937	22.38	84-74-2	Dibutyl phthalate	235845.3
110	44.58	783	871	88.1	82304-66-3	7,9-Di-tert-butyl-1-oxaspiro(4,5)deca-6,9-dier	60516.78
111	45.12	705	766	46.14	1002-84-2	Pentadecanoic acid	77383.55

WT-3

Peak	Ret.Time	match	R.match	Prob	CAS Number	Library Compound	Area
2	11.97	850	853	73.25	513-86-0	Acetoin	559464.6
3	14.31	800	877	55.11	123-32-0	Pyrazine, 2,5-dimethyl-	49840.53
10	16.26	930	955	92.3	821-55-6	2-Nonanone	693537.6
15	18.18	696	801	56.91	693-54-9	2-Decanone	161782.7
16	18.48	716	768	45.17	693-54-9	2-Decanone	151915.1
17	19.01	933	944	49.47	104-76-7	1-Hexanol, 2-ethyl-	844167.5
18	19.69	816	842	28.9	112-31-2	Decanal	225036.3
20	20.05	676	911	16.13	100-52-7	Benzaldehyde	548851.3
21	20.31	790	872	56.21	24347-58-8	2,3-Butanediol, [R-(R*,R*)]-	357374.4
24	21.21	883	903	11.87	36653-82-4	1-Hexadecanol	377967
28	22.14	894	901	83.65	112-12-9	2-Undecanone	279043.8
29	22.24	843	880	45.43	17322-97-3	1,2-Epoxyundecane	389105
34	23.42	789	811	40.56	6175-49-1	2-Dodecanone	1138760
36	23.7	812	813	53.65	6175-49-1	2-Dodecanone	325140.3
37	23.8	754	884	5.53	1653-30-1	2-Undecanol	101488.8
38	24.63	929	959	44.36	112-54-9	Dodecanal	14331080
39	25.24	824	877	32.66	275-51-4	Azulene	199130
40	25.69	883	907	14.36	112-30-1	1-Decanol	257272
41	25.8	827	841	88.26	0	Oxime-, methoxy-phenyl-_	218710.5
43	26.03	827	859	7.63	74962-98-4	2-Tridecen-1-ol, (E)-	210154.6
44	26.1	835	860	22	10203-28-8	2-Dodecanol	373434.7
45	26.38	825	829	8.69	3234-28-4	Oxirane, dodecyl-	1565202
46	26.97	800	887	56.68	593-08-8	2-Tridecanone	726355.4
50	27.96	796	881	9.34	112-42-5	1-Undecanol	68947.85
51	28.12	831	848	50.89	2345-27-9	2-Tetradecanone	763332.9
53	28.41	816	826	37.04	2345-27-9	2-Tetradecanone	802685.4
55	28.73	903	915	1900/3/24	60-12-8	Phenylethyl Alcohol	360270.2
58	29.16	822	880	31.24	1653-31-2	2-Tridecanol	208460
60	29.57	807	831	54.03	2146-71-6	Dodecanoic acid, ethenyl ester	69712.82
62	29.83	745	896	49.6	272-16-2	1,2-Benzisothiazole	28551.77
64	30.01	923	924	9	112-53-8	1-Dodecanol	906555.8
65	30.29	805	848	12.64	4706-81-4	2-Tetradecanol	268188.6
66	30.57	771	830	8.78	4706-81-4	2-Tetradecanol	133488.9
67	31.28	783	812	49.95	2345-28-0	2-Pentadecanone	85759.5
69	31.47	727	784	11.8	2306-78-7	Nerolidyl acetate	41727
70	32.12	714	864	28.16	106-44-5	p-Cresol	69073.23
71	32.3	855	858	63.9	18787-63-8	2-Hexadecanone	209999
73	32.59	784	865	38.15	629-66-3	2-Nonadecanone	356547.6
83	35.56	775	815	54.15	628-97-7	Hexadecanoic acid, ethyl ester	59686.69
89	36.24	908	910	55.85	96-76-4	2,4-Di-tert-butylphenol	362975.1
90	37.06	789	855	23.14	3790-71-4	2,6,10-Dodecatrien-1-ol, 3,7,11-trimethyl-, (Z,E)	99443.73
92	37.69	675	821	35.35	2628-17-3	4-Vinylphenol	42509
94	38.39	881	899	44.14	120-72-9	Indole	191845.4
101	43.52	936	947	24.43	84-74-2	Dibutyl phthalate	191413.2
102	44.58	704	804	73.57	82304-66-3	7,9-Di-tert-butyl-1-oxaspiro(4,5)deca-6,9-diene-2	40945.07
103	45.11	783	820	63.36	1002-84-2	Pentadecanoic acid	115131.6

HN-Q-8-ΔsrfAA-1

Peak	Ret.Time	match	R.match	Prob	CAS Number	Library Compound	Area
1	6.44	905	906	50.48	71-36-3	1-Butanol	138291.796
4	12	818	826	68.88	513-86-0	Acetoin	530275.063
5	14.26	798	862	41.8	123-32-0	Pyrazine, 2,5-dimethyl-	28380.745
9	14.48	746	866	52.57	123-32-0	Pyrazine, 2,5-dimethyl-	55766.289
10	16.26	925	939	92.09	821-55-6	2-Nonanone	740093.987
14	19.01	930	942	61.04	104-76-7	1-Hexanol, 2-ethyl-	493184.803
16	19.99	892	922	58.13	100-52-7	Benzaldehyde	607549.18
17	20.33	676	929	67.16	19132-06-0	2,3-Butanediol, [S-(R*,R*)]-	276319.21
27	23.02	750	887	50.95	122-78-1	Benzeneacetaldehyde	207724.414
29	23.33	708	735	32.21	4536-23-6	Hexanoic acid, 2-methyl-	1522612.644
30	23.65	805	824	46.79	6175-49-1	2-Dodecanone	205673.032
32	23.93	656	800	13.66	6175-49-1	2-Dodecanone	132581.008
33	24.77	890	957	47.29	112-54-9	Dodecanal	2058740.861
36	25.71	811	854	89.25	0	Oxime-, methoxy-phenyl-_	135243.84
37	25.81	820	833	86.5	0	Oxime-, methoxy-phenyl-_	834396.993
41	26.43	754	848	12.29	30689-78-2	(R)-(-)-(Z)-14-Methyl-8-hexadecen-1-ol	72920.981
42	26.48	743	834	9.02	30689-78-2	(R)-(-)-(Z)-14-Methyl-8-hexadecen-1-ol	82297.958
51	27.98	626	801	49.99	77-68-9	Propanoic acid, 2-methyl-, 3-hydroxy-2,2,4-trimethylpentyl	41249.812
52	28.12	807	854	64.51	2345-27-9	2-Tetradecanone	953812.288
53	28.42	755	869	53.66	629-66-3	2-Nonadecanone	880682.325
55	28.75	889	899	73.81	60-12-8	Phenylethyl Alcohol	354834.403
60	29.86	740	880	66.44	95-16-9	Benzothiazole	52971.591
61	30.01	911	915	11.32	74663-85-7	Cyclopropane, nonyl-	528217.49
66	30.61	682	882	26.79	108-95-2	Phenol	46145.115
67	31.23	914	918	83.1	2345-28-0	2-Pentadecanone	335526.009
70	32.13	712	849	21.61	106-44-5	p-Cresol	55617.092
71	32.26	872	874	64.32	18787-63-8	2-Hexadecanone	620556.365
81	34.07	799	870	11.72	4706-81-4	2-Tetradecanol	95948.208
83	34.87	757	833	23.59	5129-60-2	Pentadecanoic acid, 14-methyl-, methyl ester	119990.471
86	35.14	756	862	18.63	629-66-3	2-Nonadecanone	108510.572
88	35.54	862	872	72.65	628-97-7	Hexadecanoic acid, ethyl ester	165911.912
91	36.22	907	907	58.37	96-76-4	2,4-Di-tert-butylphenol	487878.206
102	37.53	797	913	6.36	36653-82-4	1-Hexadecanol	81669.056
104	38.38	739	855	26.34	120-72-9	Indole	59080.751
107	38.97	643	775	24.01	111-61-5	Octadecanoic acid, ethyl ester	32481.452
119	43.5	928	939	18.45	84-74-2	Dibutyl phthalate	455400.802
121	44.56	777	868	80.1	82304-66-3	7,9-Di-tert-butyl-1-oxaspiro(4,5)deca-6,9-diene-2,8-dione	91101.114
126	45.78	692	724	46.3	0	i-Propyl 12-methyltetradecanoate	115940.762
127	46.88	773	810	59.33	1002-84-2	Pentadecanoic acid	101338.783

HN-Q-8-ΔsrfAAΔbaeBE-2

Peak	Ret.Time	match	R.match	Prob	CAS Number	Library Compound	Area
2	8.59	583	793	19.51	123-51-3	1-Butanol, 3-methyl-	11147.63
4	8.69	697	833	22.59	71-41-0	1-Pentanol	30200.03
6	11.89	856	859	73.76	513-86-0	Acetoin	1918529
7	16.35	923	931	91.08	821-55-6	2-Nonanone	582238.1
10	18.04	682	918	59.43	540-73-8	Hydrazine, 1,2-dimethyl-	36054.26
13	19.07	920	932	46.62	104-76-7	1-Hexanol, 2-ethyl-	427009.9
18	20.27	886	915	43.8	19132-06-0	2,3-Butanediol, [S-(R*,R*)]-	1806974
28	23.19	615	867	64.16	24295-03-2	2-Acetylthiazole	104979.4
30	23.66	754	820	41.84	6175-49-1	2-Dodecanone	77745.43
32	23.94	715	796	16.18	6175-49-1	2-Dodecanone	94939.43
33	24.79	906	949	43.64	112-54-9	Dodecanal	2067952
34	25.06	748	849	51.53	103-69-5	Benzenamine, N-ethyl-	182101.6
36	25.81	825	865	84.22	0	Oxime-, methoxy-phenyl-_	611971.3
40	26.51	756	834	8.1	30689-78-2	(R)-(-)-(Z)-14-Methyl-8-hexadecen-1-ol	74007.79
45	27.75	665	897	20.18	17372-78-0	Cinnoline, 3-methyl-	49198.18
48	28.08	864	891	29.22	100-51-6	Benzyl alcohol	119433.3
49	28.19	741	828	48.82	2345-27-9	2-Tetradecanone	102954.3
51	28.48	661	802	34.31	629-66-3	2-Nonadecanone	307349.2
52	28.75	900	917	########	60-12-8	Phenylethyl Alcohol	249747.4
53	29.11	868	940	26.22	10468-64-1	Benzene, 1-isocyano-2-methyl-	234003.7
55	30.02	912	937	9.08	112-42-5	1-Undecanol	830780.3
56	30.35	727	806	35.57	2345-28-0	2-Pentadecanone	93798.74
58	31.31	767	808	57.43	2345-28-0	2-Pentadecanone	77309.89
62	32.36	744	831	22.06	6175-49-1	2-Dodecanone	50402.12
64	32.61	738	869	36.21	629-66-3	2-Nonadecanone	126426.2
66	33.96	805	891	6.82	36653-82-4	1-Hexadecanol	95389.8
69	34.89	674	744	24.83	112-39-0	Hexadecanoic acid, methyl ester	56252.71
73	35.57	828	855	65.77	628-97-7	Hexadecanoic acid, ethyl ester	57906.82
75	36.23	914	914	58.08	96-76-4	2,4-Di-tert-butylphenol	1369547
78	37.08	747	823	11.35	4602-84-0	2,6,10-Dodecatrien-1-ol, 3,7,11-trimethyl-	45718.84
79	37.3	652	808	13.83	84-66-2	Diethyl Phthalate	24112.33
80	37.54	758	863	3.48	36653-82-4	1-Hexadecanol	48381.49
82	38.42	689	828	19.66	120-72-9	Indole	30783.6
83	38.99	579	750	18.45	111-61-5	Octadecanoic acid, ethyl ester	62119.25
87	40.19	870	889	6.33	0	Phthalic acid, hex-3-yl isobutyl ester	66708.65
90	42.38	693	793	24.36	544-63-8	Tetradecanoic acid	163996.1
91	43.52	935	946	24.08	84-74-2	Dibutyl phthalate	305498
92	44.58	694	837	79.51	82304-66-3	7,9-Di-tert-butyl-1-oxaspiro(4,5)deca-6,9-	64397.22
93	45.11	744	790	54.34	1002-84-2	Pentadecanoic acid	75377.41

HN-Q-8-ΔsrfAAΔbaeBE-1

Peak	Ret.Time	match	R.match	Prob	CAS Number	Library Compound	Area
1	8.69	812	863	57.96	123-51-3	1-Butanol, 3-methyl-	64718.69
3	11.9	851	855	67.86	513-86-0	Acetoin	1660250
4	12.39	500	852	17.48	513-86-0	Acetoin	17134.05
5	16.36	904	918	87.97	821-55-6	2-Nonanone	620907.1
6	16.87	447	764	5.59	821-55-6	2-Nonanone	15221.47
14	19.11	881	916	58	104-76-7	1-Hexanol, 2-ethyl-	164903
16	20.29	889	917	48.33	24347-58-8	2,3-Butanediol, [R-(R*,R*)]-	1334903
26	23.2	597	896	70.05	24295-03-2	2-Acetylthiazole	122106.1
27	23.51	746	778	8.68	143-08-8	1-Nonanol	307372.2
28	23.67	700	789	42.5	6175-49-1	2-Dodecanone	96978.17
30	23.94	730	816	34.5	6175-49-1	2-Dodecanone	104021.2
31	24.8	904	956	46.37	112-54-9	Dodecanal	2407987
33	25.83	825	867	83.49	0	Oxime-, methoxy-phenyl-_	693834.3
36	26.51	788	845	11.42	30689-78-2	(R)-(-)-(Z)-14-Methyl-8-hexadecen-1-ol	93165.94
41	27.74	667	915	56.13	13679-41-9	Furan, 3-phenyl-	49302.44
43	28.09	889	919	45.51	100-51-6	Benzyl alcohol	97610.49
44	28.2	735	822	41.48	2345-27-9	2-Tetradecanone	100036.4
45	28.47	646	845	35.11	629-66-3	2-Nonadecanone	193293.8
47	28.76	911	926	84.06	60-12-8	Phenylethyl Alcohol	256159.1
48	29.12	863	892	########	620-22-4	Benzonitrile, 3-methyl-	228864
50	30.04	922	926	9.03	112-53-8	1-Dodecanol	786538.7
51	30.36	744	797	28.76	2345-28-0	2-Pentadecanone	90860.1
54	31.31	782	825	52.96	2345-28-0	2-Pentadecanone	69830.94
57	32.36	785	833	63.47	18787-63-8	2-Hexadecanone	60566.35
59	32.62	725	846	27.08	629-66-3	2-Nonadecanone	165020.3
66	35.58	836	859	70.47	628-97-7	Hexadecanoic acid, ethyl ester	68209.36
69	36.23	907	907	56.87	96-76-4	2,4-Di-tert-butylphenol	1379317
71	37.08	741	800	9.22	4602-84-0	2,6,10-Dodecatrien-1-ol, 3,7,11-trimet	48920.48
72	37.31	614	806	15.38	84-66-2	Diethyl Phthalate	16887.45
76	38.41	708	844	21.5	120-72-9	Indole	39355.43
81	40.19	902	916	9.88	84-69-5	1,2-Benzenedicarboxylic acid, bis(2-me	63273.96
84	42.39	648	780	26.88	544-63-8	Tetradecanoic acid	102023
85	43.52	929	932	22.87	84-74-2	Dibutyl phthalate	230040.6
87	44.6	700	800	74.92	82304-66-3	7,9-Di-tert-butyl-1-oxaspiro(4,5)deca-	30412.33

HN-Q-8-ΔbaeBE-3

Peak	Ret.Time	match	R.match	Prob	CAS Number	Library Compound	Area
2	11.9	833	837	75.14	513-86-0	Acetoin	2716667
3	15.11	624	758	30.84	3658-80-8	Dimethyl trisulfide	113926.3
4	15.15	527	749	20.75	3658-80-8	Dimethyl trisulfide	86240.92
5	16.36	913	928	90.87	821-55-6	2-Nonanone	381402.4
10	19.07	925	938	57.95	104-76-7	1-Hexanol, 2-ethyl-	351955.7
12	20.21	777	881	29.59	100-52-7	Benzaldehyde	84927.66
13	20.31	855	885	42.8	24347-58-8	2,3-Butanediol, [R-(R*,R*)]-	781489.2
17	21.01	842	922	35	111-87-5	1-Octanol	79394.26
25	21.6	716	824	12.18	30689-78-2	(R)-(-)-(Z)-14-Methyl-8-hexadecen-1-ol	47302.28
27	22.25	899	910	88.37	112-12-9	2-Undecanone	227008.8
29	22.46	608	837	35.38	17322-97-3	1,2-Epoxyundecane	258334.3
32	23.18	645	877	75.55	24295-03-2	2-Acetylthiazole	100491.9
33	23.47	752	779	7.82	143-08-8	1-Nonanol	202088.5
34	23.59	779	828	62.31	6175-49-1	2-Dodecanone	73924.69
36	23.86	819	840	41.45	6175-49-1	2-Dodecanone	138696.5
37	24.74	913	959	42.32	112-54-9	Dodecanal	4433795
39	25.26	608	923	16.95	2471-84-3	1H-Indene, 1-methylene-	121596.3
40	25.73	784	892	7.58	112-30-1	1-Decanol	258839.6
41	25.82	837	851	91.55	0	Oxime-, methoxy-phenyl-_	739596.3
42	26.13	643	837	19.99	3234-28-4	Oxirane, dodecyl-	78125.62
44	26.44	801	822	10.01	69064-37-5	trans-2-Dodecen-1-ol	272505.5
52	28	794	849	25.03	0	Undecyl butyrate	115290.8
53	28.09	809	877	36.77	100-51-6	Benzyl alcohol	52200.61
54	28.19	622	776	24.25	2345-27-9	2-Tetradecanone	187781.9
56	28.45	691	780	41.23	0	Pentanoic acid, 2,2,4-trimethyl-3-carboxyis	797970.2
57	28.78	900	924	########	60-12-8	Phenylethyl Alcohol	199869
60	29.84	789	875	42.24	272-16-2	1,2-Benzisothiazole	73168.9
61	30.03	919	920	7.51	112-53-8	1-Dodecanol	666297.3
63	30.36	784	823	50.85	2345-28-0	2-Pentadecanone	78322.58
64	31.3	791	816	62.14	2345-28-0	2-Pentadecanone	105410
66	31.86	660	772	28.31	124-06-1	Tetradecanoic acid, ethyl ester	44833.62
67	32.35	783	834	52.89	18787-63-8	2-Hexadecanone	92916.01
69	32.63	756	813	36.78	18787-63-8	2-Hexadecanone	166349.9
71	34.88	647	807	24.59	5129-60-2	Pentadecanoic acid, 14-methyl-, methyl este	70161.59
74	35.57	830	853	69.3	628-97-7	Hexadecanoic acid, ethyl ester	68241.63
81	36.24	901	901	53.89	96-76-4	2,4-Di-tert-butylphenol	507345.3
90	37.08	759	861	18.01	3790-71-4	2,6,10-Dodecatrien-1-ol, 3,7,11-trimethyl-,	56072.83
91	37.31	668	822	16.75	84-66-2	Diethyl Phthalate	28714.6
92	37.54	688	856	2.73	36653-82-4	1-Hexadecanol	31273.03
104	42.38	731	796	53.7	544-63-8	Tetradecanoic acid	147870.1
105	43.54	924	936	16.13	84-74-2	Dibutyl phthalate	328512.3
106	45.13	643	780	25.29	1002-84-2	Pentadecanoic acid	52682.28
107	45.64	701	722	46.25	0	i-Propyl 12-methyltetradecanoate	178264.4

HN-Q-8-ΔbaeBE-2

Peak	Ret.Time	match	R.match	Prob	CAS Number	Library Compound	Area
2	11.91	829	833	70.24	513-86-0	Acetoin	2508931
3	16.3	931	954	91.61	821-55-6	2-Nonanone	498841.7
12	19.03	931	947	63.76	104-76-7	1-Hexanol, 2-ethyl-	376043
15	20.18	807	905	55.93	100-52-7	Benzaldehyde	155571.2
16	20.3	893	925	62.36	24347-58-8	2,3-Butanediol, [R-(R*,R*)]-	557435.6
18	20.98	767	885	22.09	2511-91-3	Cyclopropane, pentyl-	39189.16
19	21.23	784	851	12.08	112-44-7	Undecanal	117335.6
25	22.21	838	869	81.31	112-12-9	2-Undecanone	146857.5
29	23.44	793	864	14.06	112-54-9	Dodecanal	1086244
30	23.56	782	856	27.76	17322-97-3	1,2-Epoxyundecane	710507.6
31	23.84	756	800	12.89	6175-49-1	2-Dodecanone	216892.7
33	24.47	829	902	19.43	112-54-9	Dodecanal	257385.3
34	24.7	805	941	27.99	112-54-9	Dodecanal	4676314
35	25.31	779	833	77.17	0	Oxime-, methoxy-phenyl-_	249766.9
37	25.81	767	836	83.13	0	Oxime-, methoxy-phenyl-_	784864.4
40	26.42	777	822	8.1	30689-78-2	(R)-(-)-(Z)-14-Methyl-8-hexadecen-1-ol	241112.6
47	27.98	756	830	11.79	0	Undecyl butyrate	104366.6
50	28.76	895	905	78.16	60-12-8	Phenylethyl Alcohol	272446.6
54	29.61	656	847	33.65	272-16-2	1,2-Benzisothiazole	16258.09
55	29.63	518	850	8.58	272-16-2	1,2-Benzisothiazole	34888.19
56	29.8	805	854	63.34	95-16-9	Benzothiazole	87149.84
57	29.99	923	948	13.48	112-42-5	1-Undecanol	1092847
59	30.31	834	859	63.34	2345-28-0	2-Pentadecanone	172283.2
63	31.24	879	883	74.31	2345-28-0	2-Pentadecanone	202755.5
65	31.79	696	778	29.14	124-06-1	Tetradecanoic acid, ethyl ester	61375.88
69	32.28	844	855	61.71	18787-63-8	2-Hexadecanone	193941.8
71	32.57	737	875	45.47	629-66-3	2-Nonadecanone	316063.6
77	33.93	789	902	3.91	112-42-5	1-Undecanol	118002.7
80	34.85	707	756	37.51	112-39-0	Hexadecanoic acid, methyl ester	122058.3
86	35.54	861	868	77.25	628-97-7	Hexadecanoic acid, ethyl ester	204916.9
93	36.08	713	869	45.19	131-11-3	Dimethyl phthalate	49962.29
95	36.23	919	919	58.54	96-76-4	2,4-Di-tert-butylphenol	769623.5
96	37.05	794	855	19.21	3790-71-4	2,6,10-Dodecatrien-1-ol, 3,7,11-trimethyl-, (	89411.38
97	37.28	778	873	35.27	84-66-2	Diethyl Phthalate	89080.64
98	37.52	797	914	13.16	36653-82-4	1-Hexadecanol	79858.19
100	38.4	770	867	31.1	120-72-9	Indole	82906.38
106	40.18	884	888	6.72	84-69-5	1,2-Benzenedicarboxylic acid, bis(2-methylprop	146962.2
110	42.34	742	815	50.88	544-63-8	Tetradecanoic acid	171504.9
111	43.5	944	945	27.32	84-74-2	Dibutyl phthalate	630738.4
112	44.58	744	846	79.83	82304-66-3	7,9-Di-tert-butyl-1-oxaspiro(4,5)deca-6,9-dien	60693.97
113	45.08	812	842	63.7	1002-84-2	Pentadecanoic acid	126545.5

HN-Q-8-ΔbaeBE-1

Peak	Ret.Time	match	R.match	Prob	CAS Number	Library Compound	Area
2	11.84	820	823	68.52	513-86-0	Acetoin	2422877.095
3	16.85	755	886	63.11	821-55-6	2-Nonanone	66085.401
14	19.04	866	891	90.49	1124-11-4	Pyrazine, tetramethyl-	248215.324
15	19.2	636	797	15.2	58175-57-8	2-Propyl-1-pentanol	513341.264
17	20.12	927	933	64.98	100-52-7	Benzaldehyde	349828.739
18	20.32	677	830	38.7	24347-58-8	2,3-Butanediol, [R-(R*,R*)]-	604169.594
26	23.22	569	902	57.44	24295-03-2	2-Acetylthiazole	59275.328
27	23.66	701	840	11.34	5451-96-7	Chloroacetic acid, nonyl ester	154069.64
29	24.99	938	949	44.67	112-54-9	Dodecanal	1531861.559
33	25.82	785	834	75.87	0	Oxime-, methoxy-phenyl-_	64459.759
34	25.87	769	884	7.42	5451-52-5	Formic acid, decyl ester	172200.783
36	27.29	824	880	25.52	15764-16-6	Benzaldehyde, 2,4-dimethyl-	48788.068
47	28.76	870	928	########	60-12-8	Phenylethyl Alcohol	433169.393
52	29.88	804	921	69.41	272-16-2	1,2-Benzisothiazole	63191.977
54	30.17	745	919	10.2	112-42-5	1-Undecanol	311409.063
55	30.59	644	845	18.97	108-95-2	Phenol	73688.494
66	36.23	899	902	52.9	96-76-4	2,4-Di-tert-butylphenol	346491.156
67	37.16	673	776	7.85	4602-84-0	2,6,10-Dodecatrien-1-ol, 3,7,11-trimethyl-	28265.127
71	38.38	765	890	30.15	120-72-9	Indole	66738.855
73	40.27	880	882	7.92	84-69-5	1,2-Benzenedicarboxylic acid, bis(2-methylpropyl) ester	159182.212
75	43.65	894	907	14.95	84-74-2	Dibutyl phthalate	137528.699
79	36.09	546	831	14.6	629-66-3	2-Nonadecanone	92325.295
80	36.22	900	900	54.92	96-76-4	2,4-Di-tert-butylphenol	1932161.081
82	37.51	751	904	7.82	36653-82-4	1-Hexadecanol	102400.604
84	38.39	786	886	32.77	120-72-9	Indole	79998.267
98	42.38	652	782	29.95	544-63-8	Tetradecanoic acid	74641.44
100	43.49	938	940	17	84-74-2	Dibutyl phthalate	351100.444
102	44.56	821	881	88.9	82304-66-3	7,9-Di-tert-butyl-1-oxaspiro(4,5)deca-6,9-diene-2,8-dione	98871.389
103	45.08	814	841	58.06	1002-84-2	Pentadecanoic acid	202472.577

HN-Q-8-ΔsrfAA-3

Peak	Ret.Time	match	R.match	Prob	CAS Number	Library Compound	Area
1	6.49	874	877	47.83	71-36-3	1-Butanol	217653.9
3	8.74	869	900	63.3	123-51-3	1-Butanol, 3-methyl-	50510.83
5	12	852	854	82.36	513-86-0	Acetoin	827241.5
6	14.25	880	895	66.84	123-32-0	Pyrazine, 2,5-dimethyl-	175502.1
7	16.22	927	945	91.64	821-55-6	2-Nonanone	839898.2
11	18.31	699	946	28.33	40575-42-6	1-Octen-4-ol	58992.29
14	19.01	932	940	64.13	104-76-7	1-Hexanol, 2-ethyl-	605858.9
16	19.99	907	917	56.86	100-52-7	Benzaldehyde	575085.5
17	20.31	733	894	51.69	19132-06-0	2,3-Butanediol, [S-(R*,R*)]-	288743.6
19	20.93	777	906	69.44	79-31-2	Propanoic acid, 2-methyl-	65424.78
26	23.13	594	890	66.44	24295-03-2	2-Acetylthiazole	134528.8
27	23.32	694	742	27.33	116-53-0	Butanoic acid, 2-methyl-	1819944
28	23.64	771	813	45.3	6175-49-1	2-Dodecanone	230854.9
30	23.93	683	801	14.84	6175-49-1	2-Dodecanone	110614.4
31	24.76	862	938	32.36	112-54-9	Dodecanal	2010578
32	25.03	785	861	42.18	103-69-5	Benzenamine, N-ethyl-	275301.7
34	25.72	836	857	92.96	0	Oxime-, methoxy-phenyl-_	178867
35	25.81	823	847	90.74	0	Oxime-, methoxy-phenyl-_	1017984
36	26.12	573	816	8.86	112-44-7	Undecanal	58815.06
39	26.46	784	814	8.26	69064-37-5	trans-2-Dodecen-1-ol	209822
42	27.29	626	876	4.56	73105-67-6	1-Iodo-2-methylundecane	116295.4
49	27.99	682	805	49.49	77-68-9	Propanoic acid, 2-methyl-, 3-hydroxy-2,2,4-trim	41942.69
50	28.13	770	845	58.29	2345-27-9	2-Tetradecanone	781416.9
53	28.75	903	914	########	60-12-8	Phenylethyl Alcohol	325661.4
58	29.85	674	884	46.68	272-16-2	1,2-Benzisothiazole	23903.68
60	30.02	911	914	8.25	74663-85-7	Cyclopropane, nonyl-	585468.3
65	30.61	573	848	14.02	108-95-2	Phenol	35470.77
66	31.23	875	876	69.29	2345-28-0	2-Pentadecanone	316805.3
67	31.76	651	774	27.18	124-06-1	Tetradecanoic acid, ethyl ester	66382.35
69	32.14	700	857	29.17	106-44-5	p-Cresol	49269.01
70	32.26	868	870	61.26	18787-63-8	2-Hexadecanone	728549.5
72	32.56	791	811	33.25	18787-63-8	2-Hexadecanone	612291.4
77	33.31	666	784	33.13	502-69-2	2-Pentadecanone, 6,10,14-trimethyl-	66140.41
78	33.93	818	905	7.44	14852-31-4	2-Hexadecanol	163940.9
79	34.08	812	900	13.41	14852-31-4	2-Hexadecanol	85776.55
81	34.86	709	754	31.25	112-39-0	Hexadecanoic acid, methyl ester	101962.1
82	35.15	774	862	32.11	629-66-3	2-Nonadecanone	103515.5
84	35.56	846	860	68.26	628-97-7	Hexadecanoic acid, ethyl ester	114112.8
86	36.22	898	898	55.97	96-76-4	2,4-Di-tert-butylphenol	345706.1
97	37.53	775	894	4.87	36653-82-4	1-Hexadecanol	91841.72
100	38.4	791	872	31.1	120-72-9	Indole	64794.2
108	43.51	945	949	34.34	84-74-2	Dibutyl phthalate	362603.1
109	44.56	693	829	76.16	82304-66-3	7,9-Di-tert-butyl-1-oxaspiro(4,5)deca-6,9-diene	53842.33
110	45.13	704	777	34.1	1002-84-2	Pentadecanoic acid	88707.51

HN-Q-8-ΔsrfAA-2

Peak	Ret.Time	match	.matc	Prob	CAS Number	Library Compound	Area
1	6.52	867	893	46.92	71-36-3	1-Butanol	269987.808
3	8.74	901	913	53.67	123-51-3	1-Butanol, 3-methyl-	63301.847
5	11.98	881	884	88.5	513-86-0	Acetoin	891139.721
6	14.23	835	884	67.44	123-32-0	Pyrazine, 2,5-dimethyl-	48253.862
7	14.31	659	853	53.41	123-32-0	Pyrazine, 2,5-dimethyl-	65499.449
8	14.48	734	868	61.43	123-32-0	Pyrazine, 2,5-dimethyl-	52788.144
9	16.28	930	961	91.48	821-55-6	2-Nonanone	731009.78
10	16.7	551	714	8.74	29461-03-8	Pyrazine, 2-methyl-5-propyl-	20118.948
13	18.31	571	835	8.73	19549-79-2	4-Heptanol, 3,5-dimethyl-	97461.951
15	19.02	935	947	66.99	104-76-7	1-Hexanol, 2-ethyl-	443828.824
18	20.03	914	932	62.84	100-52-7	Benzaldehyde	614045.805
19	20.35	671	836	48.67	24347-58-8	2,3-Butanediol, [R-(R*,R*)]-	280084.767
28	23.13	542	814	34.08	24295-03-2	2-Acetylthiazole	160079.903
29	23.34	916	924	77.56	98-00-0	2-Furanmethanol	335808.294
31	23.66	846	864	60.94	6175-49-1	2-Dodecanone	121048.463
34	24.78	868	933	34.89	112-54-9	Dodecanal	2055833.42
36	25.71	822	857	88.71	0	Oxime-, methoxy-phenyl-_	169700.741
37	25.81	830	850	90.56	0	Oxime-, methoxy-phenyl-_	918790.736
40	26.46	781	849	16.19	30689-78-2	(R)-(-)-(Z)-14-Methyl-8-hexadecen-1-ol	134427.463
46	28.06	945	951	57.19	100-51-6	Benzyl alcohol	137269.677
47	28.14	812	845	65.2	2345-27-9	2-Tetradecanone	578953.175
49	28.74	897	915	1900/3/18	60-12-8	Phenylethyl Alcohol	337776.352
51	29.17	693	917	59.13	20600-54-8	Benzene, 1-isocyano-3-methyl-	57149.072
54	29.82	790	895	48.43	272-16-2	1,2-Benzisothiazole	60068.163
55	30.03	915	918	11.05	74663-85-7	Cyclopropane, nonyl-	397798.55
58	30.63	622	854	16.49	108-95-2	Phenol	49379.155
59	31.23	860	860	67.38	2345-28-0	2-Pentadecanone	236206.092
63	32.13	765	886	27.56	106-44-5	p-Cresol	37085.218
64	32.25	853	856	62.61	18787-63-8	2-Hexadecanone	653126.422
68	33.93	814	895	6.96	36653-82-4	1-Hexadecanol	124207.615
72	34.87	806	850	43.99	112-39-0	Hexadecanoic acid, methyl ester	91026.108
74	35.15	747	840	16.46	629-66-3	2-Nonadecanone	95974.45
76	35.54	857	864	72.71	628-97-7	Hexadecanoic acid, ethyl ester	117947.303
80	36.22	900	900	54.92	96-76-4	2,4-Di-tert-butylphenol	1932161.081
82	37.51	751	904	7.82	36653-82-4	1-Hexadecanol	102400.604
84	38.39	786	886	32.77	120-72-9	Indole	79998.267
98	42.38	652	782	29.95	544-63-8	Tetradecanoic acid	74641.44
100	43.49	938	940	17	84-74-2	Dibutyl phthalate	351100.444
102	44.56	821	881	88.9	82304-66-3	7,9-Di-tert-butyl-1-oxaspiro(4,5)deca-6,9-diene-2,8-dione	98871.389
103	45.08	814	841	58.06	1002-84-2	Pentadecanoic acid	202472.577

HN-Q-8-ΔsrfAAΔbaeBE-3

Peak	Ret.Time	match	R.match	Prob	CAS Number	Library Compound	Area
1	8.68	809	828	34.49	123-51-3	1-Butanol, 3-methyl-	109315.8
5	11.89	836	840	72.28	513-86-0	Acetoin	1856926
6	16.37	933	947	91.64	821-55-6	2-Nonanone	641411.9
8	18.06	585	934	40.56	540-73-8	Hydrazine, 1,2-dimethyl-	20587.41
15	19.09	882	915	35.71	104-76-7	1-Hexanol, 2-ethyl-	219533.1
17	20.27	849	877	45.55	513-85-9	2,3-Butanediol	1297163
20	21.03	736	899	26.59	111-87-5	1-Octanol	42092.7
25	23.19	664	893	77.99	24295-03-2	2-Acetylthiazole	115695.5
26	23.49	618	828	4.73	7423-69-0	1-Hexene, 3,5-dimethyl-	228864.6
27	23.63	742	800	49.52	6175-49-1	2-Dodecanone	83779.11
30	23.91	761	823	31.03	6175-49-1	2-Dodecanone	97277.86
31	24.75	853	955	36.92	112-54-9	Dodecanal	2819980
33	25.74	895	901	8	112-30-1	1-Decanol	183927
34	25.82	819	855	90.64	0	Oxime-, methoxy-phenyl-_	367547.1
39	26.46	758	815	7.72	74962-98-4	2-Tridecen-1-ol, (E)-	81528.9
47	28.08	882	921	44.88	100-51-6	Benzyl alcohol	94795.92
48	28.17	710	811	35.2	2345-27-9	2-Tetradecanone	227010.5
49	28.43	748	809	65.04	0	Pentanoic acid, 2,2,4-trimethyl-3-carboxyisopropyl,	746752.7
51	28.75	903	916	########	60-12-8	Phenylethyl Alcohol	286919.6
53	29.09	722	820	19.06	3682-17-5	Benzenepropanoic acid, α-(hydroxyimino)-	391851.5
55	30.02	927	929	9.07	112-53-8	1-Dodecanol	836077.3
56	30.33	729	771	24.83	2345-28-0	2-Pentadecanone	105531.5
58	31.29	783	824	57.17	2345-28-0	2-Pentadecanone	85051.51
60	31.68	579	734	12.25	0	i-Propyl 12-methyl-tridecanoate	17366.48
61	31.83	623	748	23.51	124-06-1	Tetradecanoic acid, ethyl ester	22330.41
62	32.32	794	835	35.58	18787-63-8	2-Hexadecanone	111683.9
64	32.62	773	886	36.79	629-66-3	2-Nonadecanone	214251.5
70	33.94	851	921	14.53	36653-82-4	1-Hexadecanol	98946.89
73	34.88	673	821	23.66	5129-60-2	Pentadecanoic acid, 14-methyl-, methyl ester	80389.1
76	35.56	825	850	72.76	628-97-7	Hexadecanoic acid, ethyl ester	66198.92
79	36.23	895	895	62.44	96-76-4	2,4-Di-tert-butylphenol	355987.3
86	37.06	712	827	13.14	3790-71-4	2,6,10-Dodecatrien-1-ol, 3,7,11-trimethyl-, (Z,E)-	61413.83
87	37.29	658	837	17.83	84-66-2	Diethyl Phthalate	27148.42
88	37.55	651	712	2.84	2490-48-4	1-Hexadecanol, 2-methyl-	63033.73
90	38.41	709	847	20.16	274-40-8	Indolizine	50967.99
91	39	515	727	23.33	111-61-5	Octadecanoic acid, ethyl ester	91210.51
95	40.77	654	830	27.16	18919-94-3	Tetracosamethyl-cyclododecasiloxane	144056.1
96	42.35	733	813	45.82	544-63-8	Tetradecanoic acid	197207.6
97	43.52	897	907	9	84-74-2	Dibutyl phthalate	216549.2
99	44.6	723	848	72.79	82304-66-3	7,9-Di-tert-butyl-1-oxaspiro(4,5)deca-6,9-diene-2,8-	55520.73
100	45.08	749	788	53.25	1002-84-2	Pentadecanoic acid	90976.23
